# Recent Evolutionary History of Tigers Highlights Contrasting Roles of Genetic Drift and Selection

**DOI:** 10.1093/molbev/msab032

**Published:** 2021-02-16

**Authors:** Ellie E Armstrong, Anubhab Khan, Ryan W Taylor, Alexandre Gouy, Gili Greenbaum, Alexandre Thiéry, Jonathan T Kang, Sergio A Redondo, Stefan Prost, Gregory Barsh, Christopher Kaelin, Sameer Phalke, Anup Chugani, Martin Gilbert, Dale Miquelle, Arun Zachariah, Udayan Borthakur, Anuradha Reddy, Edward Louis, Oliver A Ryder, Yadvendradev V Jhala, Dmitri Petrov, Laurent Excoffier, Elizabeth Hadly, Uma Ramakrishnan

**Affiliations:** 1 Department of Biology, Stanford University, Stanford, CA, USA; 2 National Centre for Biological Sciences, TIFR, Bangalore, India; 3 End2End Genomics, LLC, Davis, CA, USA; 4 Institute of Ecology and Evolution, University of Bern, Bern, Switzerland; 5 Swiss Institute of Bioinformatics, Lausanne, Switzerland; 6 Department of Ecology, Evolution & Behavior, The Hebrew University of Jerusalem, Jerusalem, Israel; 7 Genome Institute of Singapore, A*STAR, Singapore; 8 Hudsonalpha Institute, Hunstville, AL, USA; 9 Department of Genetics, Stanford University, Stanford, CA, USA; 10 Medgenome Labs Limited, Bangalore, India; 11 Wildlife Conservation Society, Russia Program, New York, NY, USA; 12 College of Veterinary Medicine, Cornell University, Ithaca, NY, USA; 13 Kerala Forest Department, Waynad, India; 14 Aranyak, Guwahati, India; 15 Laboratory for Conservation of Endangered Species, CCMB, Hyderabad, India; 16 Department of Genetics, Omaha Zoo, Omaha, NE, USA; 17 San Diego Zoo, Institute for Conservation Research, Escondido, CA, USA; 18 Wildlife Institute of India, Dehradun, India

**Keywords:** conservation genomics, carnivores, population decline

## Abstract

Species conservation can be improved by knowledge of evolutionary and genetic history. Tigers are among the most charismatic of endangered species and garner significant conservation attention. However, their evolutionary history and genomic variation remain poorly known, especially for Indian tigers. With 70% of the world’s wild tigers living in India, such knowledge is critical. We re-sequenced 65 individual tiger genomes representing most extant subspecies with a specific focus on tigers from India. As suggested by earlier studies, we found strong genetic differentiation between the putative tiger subspecies. Despite high total genomic diversity in India, individual tigers host longer runs of homozygosity, potentially suggesting recent inbreeding or founding events, possibly due to small and fragmented protected areas. We suggest the impacts of ongoing connectivity loss on inbreeding and persistence of Indian tigers be closely monitored. Surprisingly, demographic models suggest recent divergence (within the last 20,000 years) between subspecies and strong population bottlenecks. Amur tiger genomes revealed the strongest signals of selection related to metabolic adaptation to cold, whereas Sumatran tigers show evidence of weak selection for genes involved in body size regulation. We recommend detailed investigation of local adaptation in Amur and Sumatran tigers prior to initiating genetic rescue.

## Introduction

Empirical, theoretical, and experimental studies suggest that individual and population survival is contingent on genetic variability (e.g., Saccheri et al. 1998). For endangered species that are characterized by long-term decline, small and fragmented populations, and unique selection pressures, populations may be characterized by low, but distinct standing genetic variation. Such distinct variation could result in differential probabilities of survival. Recent advances in sequencing technology, development of population genomic models, and improved computing power have revolutionized our ability to read and interpret genomes, allowing quantification of the sum total of genetic variation within individuals and populations.

For several endangered species, whole genome re-sequencing has revealed low species-level variation (e.g., Iberian lynx, Abascal et al. 2016), strong signatures of population decline (e.g., mountain gorillas: Xue et al. 2015), and recent inbreeding in isolated populations (e.g., wolves: Kardos et al. 2018). Initial studies typically sequence high-coverage genomes of a few individuals, often from ex situ collections or voucher specimens. Sampling several extant populations and larger geographic scales is often challenging (but required) for endangered species whose range spans several countries.

The tiger (*Panthera tigris*) is an iconic and charismatic endangered species that once spanned 70 degrees of latitude across Asia. It is estimated that between 2,154 and 3,159 tigers remain, which occupy less than 6% of their 1900 AD range (Goodrich et al. 2015). Despite this recent range collapse, tigers are present across 11 Asian nations, occupying diverse habitats including estuarine mangrove forests (the Sundarbans), dry deciduous forests (parts of India), tropical rainforests (Malay Peninsula), and cold, temperate forests (Russian Far East). However, the specific adaptations of the various populations to their habitats remain largely unknown.

Tigers have been classified into four extant (and four extinct) subspecies (Nowell and Jackson 1996). However, genetic and other data have suggested six (e.g., Luo et al. 2004) or two (Wilting et al. 2015) subspecies/distinct populations. Liu et al. (2018) presented the first analyses of genome-wide variation using voucher specimens across tiger range, and their data and analyses strongly supported the antiquity and uniqueness of six extant subspecies. They inferred relatively old divergences (∼68,000 years ago) between subspecies with low subsequent gene flow (1–10%) and signatures of selection in Sumatran individuals. However, their sampling of the most populous (Jhala et al. 2015) and genetically diverse tiger subspecies—the Bengal tiger—was limited across habitats.

Here, we emphasize sampling Bengal tigers from various habitats and geographic locations within India and include three other subspecies found in the wild. We use these genomes to infer historical and recent evolutionary history of tigers by investigating 1) population clustering within sampled populations, 2) genomic variation, 3) possible signatures of recent inbreeding, and 4) demographic history and differential selection. Such an approach can provide insights on future evolutionary trajectories of tiger populations.

## Results and Discussion

We used the 10× Genomics Chromium library preparation and Supernova assembler to de novo assemble a tiger genome. Based on Assemblathon2 statistics (Bradnam et al. 2013), this improved assembly corresponded to a 3.5-fold increase in the contig N50 value to 1.8 Mb and a 2.5-fold increase in the scaffold N50 value to 21.3 Mb (as compared with Cho et al. [2013]; supplementary table 2, Supplementary Material online). In addition, the resulting assembly had ∼1% fewer ambiguous bases across all scaffolds (supplementary table 2, Supplementary Material online). We also looked at gene completeness using BUSCOv4 (Simão et al. 2015), which examines highly conserved orthologs. The new assembly resulted in an 8.2% increase in the number of BUSCOs found as well as a ∼3% reduction in the number of fragmented BUSCOs and a ∼5% reduction in the number of missing BUSCOs. Repeat analysis yielded similar percentages of total repetitive content (Maltig1.0: 41.71%, Pantig1.0: 40.12%) and a slight increase in the number of annotated genes (Maltig1.0: 19,950, Pantig1.0: 19,000). Overall, the new assembly yielded a substantially more contiguous and more complete genome assembly as compared with the previous version.

**Fig. 1.**
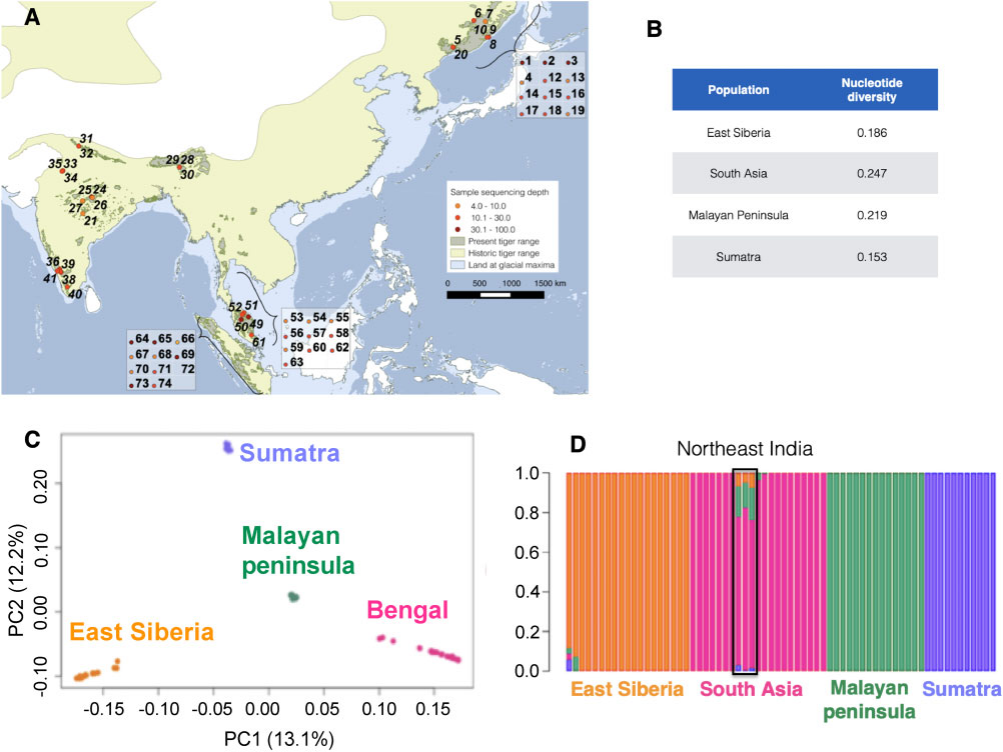
(*A*) Map of tiger samples used in this study. Each number refers to an individual. Genomic sequence coverage for each sample is color-coded. Wild samples (*n* = 32) are represented on the map, whereas captive individuals (*n* = 34) are in boxes. Sample details presented in supplementary table 1 in SI, Supplementary material online. Historical and present range map courtesy IUCN (Goodrich et al. 2015), (*B*) nucleotide diversity (pi) estimates for tigers from different regions, (*C*) principal component analyses (PCA) revealing genetic population structure in tigers and (*D*) ADMIXTURE (*K* = 4). Colors in both ADMIXTURE and PCA analyses denote individuals from the different geographical regions.

We sequenced genomes from 65 individuals (fig. 1*A*, supplementary table 1, Supplementary Material online) at varying coverage (4.2f–32.9×, median 14.4×). Our samples included wild-caught and captive-bred tigers from four putative extant sub-specific regions (South Asia, Malayan peninsula, East Siberia, and Sumatra). Details of samples used for various analyses are in supplementary table 3, Supplementary Material online. We were unable to sample the South China tiger (*P. t. amoyensis*), considered extinct-in-the-wild. Although the South China tiger is thought to be ancestral, Liu et al. (2018) suggested uncertainty about the antiquity of this population, since nuclear genomes were similar to those of Amur tigers.

### Population Structure

Both model-based (ADMIXTURE) and model-independent (PCA) analyses suggested that genetically distinct populations are concordant with earlier definitions of subspecies (as also suggested by Luo et al. [2019] and Liu et al. [2018], fig. 1*C*). We find evidence for at least four global populations based on cross-validation statistics (supplementary fig. 2, Supplementary Material online). Tigers from northeast India reveal some admixture with Malayan tigers and to a lesser extent with other subspecies (fig. 1*D*). Models with higher complexities (supplementary fig. 3, Supplementary Material online) reveal substructure within India separating south Indian tigers. However, higher model complexity fits the data poorly. In the PCA, PC1 separates the groups in a north-to-south direction whereas PC2 resolves along the east-to-west direction explaining more than 25% of the data between them (PC1: 13.1%; PC2: 12.2%).

We henceforth refer to the geographic regions by their sub-specific names (East Siberia: Amur; South Asia: Bengal; Malay Peninsula: Malayan; and Sumatra: Sumatran). Additionally, PC1 shows stronger similarity between Bengal and Malayan tigers than Bengal and Sumatran tigers, consistent with the result from *K* = 3 ADMIXTURE analyses (supplementary fig. 3, Supplementary Material online). PC2 (12.2% variation) and PC3 (10.9% variation) further separate the four groups and also separated some individuals within populations (supplementary figs. 4 and 6, Supplementary Material online). In contrast, PCA analysis of non-transcribed regions including only high-coverage individuals (coverage > 20×) within the data set (Sumatran = 3; Bengal = 3, Malayan = 3, and Amur = 3, supplementary table 6, Supplementary Material online) suggested that the Amur population is much less differentiated and closer to the Malayan population (fig. 3*B*).

PCA within subspecies (supplementary fig. 5, Supplementary Material online) suggested that Bengal tigers cluster into four sub-groups: (1) south India, (2) central and north India, (3) northeast India, and (4) northwest India. Some genomic sub-structuring was apparent in Malayan tigers, somewhat reflective of samples originating from the northern or southern Malayan peninsula (supplementary figs. 5 and 6, Supplementary Material online). Amur tigers did not demonstrate strong signatures of population sub-structuring (supplementary figs. 5 and 6, Supplementary Material online). Within subspecies, structure was confirmed in the additional PC axes for the full data set (supplementary fig. 4, Supplementary Material online). Although PC1, PC2, and PC3 separated putative subspecies (Amur, Bengal, Sumatran, and Malayan), PC1 and PC2 were also used in the supplementary figure 6*B*, Supplementary Material online, to separate the Bengal populations by geographic location clearly (northwest India, south India, and central, north, and northeast Indian tigers comprise three distinct groups).

Pairwise *F*_ST_’s (supplementary table 4, Supplementary Material online) were approximately equal between subspecies and consistent with geographic patterns. The *F*_ST_ between the Malayan and Bengal groups (0.164) was the lowest, whereas Amur and Sumatran *F*_ST_ (0.318) were highest, consistent with patterns seen in both ADMIXTURE and PCA. *F*_ST_ between putative Bengal tiger subpopulations in India (supplementary table 5Supplementary Material online) revealed high subdivision.

Since we sampled across landscapes within subspecies, we were able to compare population structuring within the four subspecies. Population genetic substructure is highest in the Indian subcontinent, whereas other tiger subspecies are genetically uniform (Amur) or less differentiated (Malayan). Our results contradict suggestions of population structure in wild Amur tigers (Sorokin et al. 2016), substantiate the significance of structure in Bengal tigers, and uncover hitherto unknown structure in tigers from the Malayan peninsula. Northeast tigers are the most distinct of Bengal tigers, although closer to Bengal tigers than to any other subspecies. The northeast Indian tigers in this study are from the state of Assam and sampling other, more eastern populations from this remote region might yield interesting insights, as would samples from Indo-Chinese tigers.

### Genetic Variation and Runs of Homozygosity

We compared genome-wide variability between tiger subspecies/subpopulations to other cats (*N* = 7) and endangered species (*N* = 8, including endangered cats). Tigers had relatively high species-level genetic diversity (supplementary fig. 7, Supplementary Material online).

Bengal tigers had the highest nucleotide diversity (pi; fig. 1*B*), whereas Sumatran tigers had the lowest. Rarefaction analysis (ADZE; Szpiech et al. 2008) revealed that diversity estimates were approaching saturation for all populations (supplementary fig. 12, Supplementary Material online). This suggests that our sampling for these populations is likely representative of the diversity levels, although more sampling would provide better resolution for some analyses.

**Fig. 2.**
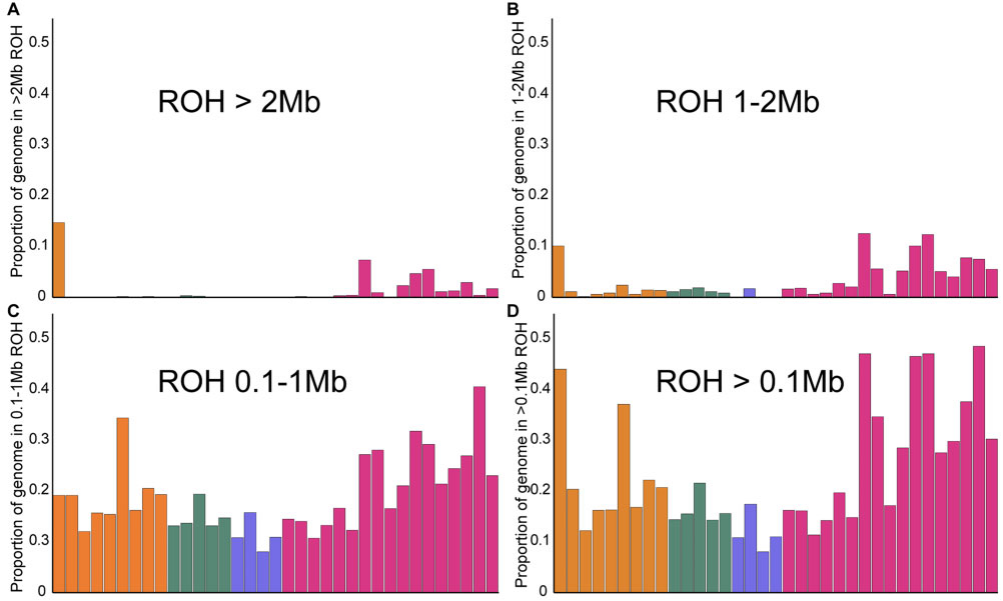
ROH inferred based on different run lengths: (*A*) > 2 Mb, (*B*) 1–2 Mb, (*C*) 100 kb–1 Mb, and (*D*) total ROH, which includes all run lengths greater than 100 kb.

Historical demography and recent inbreeding are detectable through runs of homozygosity (ROH) in the genome (Kardos et al. 2018; Pemberton et al. 2012). We quantified long (>2 Mb) and intermediate (100 kb–1 Mb, 1–2 Mb) homozygous stretches as well as the proportion of more than 100-kb-long ROH in the genome for several individuals (fig. 2). Somewhat surprisingly, individuals from the demographically large Indian tiger population revealed a high proportion of their genomes in long ROH, although variation in total ROH is high (fig. 2 and supplementary fig. 8, Supplementary Material online). Individuals from some populations (e.g., Central India) have low ROH, whereas some of the most inbred wild tigers in the world appear to be from India (e.g., from Ranthambore tiger reserve, Periyar tiger reserve, and Kaziranga tiger reserve). Results were qualitatively similar when a sliding window approach was used to estimate ROH (supplementary fig. 8, Supplementary Material online).

Our data and analyses reveal that Bengal tigers have the highest amount of variation when considering genome-wide diversity estimates. This is to be expected given historical records of Bengal tiger occupancy (Goodrich et al. 2015) across a large variety of habitats, where they subsist on a wide range of prey species that range from the large rhinoceros and gaur to the small hog deer and barking deer. Current population sizes of tigers in India and previous genetic studies based on a limited number of DNA microsatellite markers (Mondol et al. 2009) are also concordant with high genetic diversity in Bengal tigers. In contrast, certain Bengal tiger populations reveal signatures of potentially recent inbreeding/founder events or indicate population bottlenecks and isolation. High total genetic variation could reflect the large numbers of tigers prior to intense hunting in India just a century ago (Rangarajan 2006).

A comparison among populations revealed Amur tiger genomes harbor fewer long ROH than Bengal tigers. A closer look at landscapes and habitats in India and the Russian Far East reveal strong differences: India is dominated by variable habitats amidst a matrix of extremely high human population densities, whereas in the Russian Far East, human density is low, and habitat is more continuous. Indeed, landscape genetics studies have suggested that high human population density is a barrier for tiger movement (Thatte et al. 2018). We suggest that extreme fragmentation and high human population density in India have resulted in isolated populations, where individuals may be more likely to mate with relatives. In contrast, despite low Amur tiger population densities in the Russian Far East, individual movement is not hindered by significant barriers, and the population is more panmictic, with little to no sign of geographic population substructure.

The observation of high variance in long ROH in Bengal tigers underscores the importance of including genome-wide sampling across multiple individuals and within regions, as single representatives may be a poor reflection of inbreeding and variation for any given population and do not provide a context with which to evaluate significance across subspecies and populations. In the future, simulations that incorporate realistic recombination rates could be used to model and disentangle the cumulative impacts of recent demographic history and very recent inbreeding on distributions of ROH in the genome.

### Demographic History of Subspecies

We first reconstructed the past demographic history of each population with pairwise sequentially Markovian coalescent (PSMC) (supplementary fig. 9, Supplementary Material online), and our results paralleled those in Liu et al. (2018): all populations of tigers exhibit similar evolutionary patterns of population size decline.

**Fig. 3.**
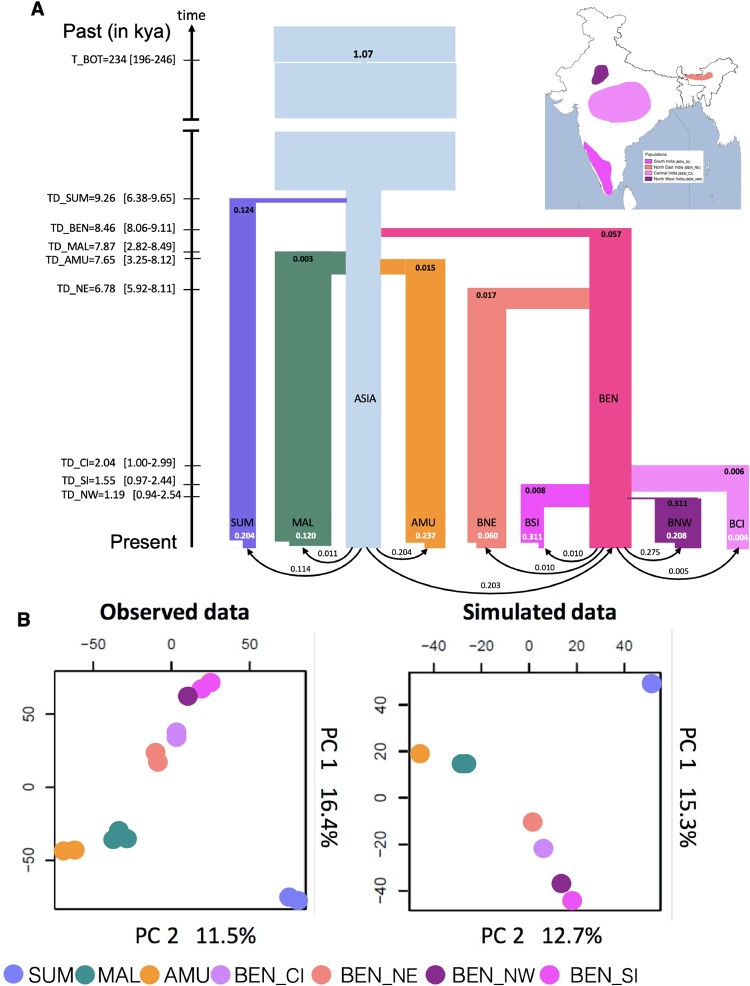
Estimated demographic history of Asian tigers: Sumatra (SUM: lavender), Malayan (MAL: dark green), Amur (AMU: orange) and ancestral Bengal (BEN: hot pink), ancestral Asian metapopulation (Asia, light blue). The Bengal tigers further differentiated into North East (BEN_NE, salmon pink), Central (BEN_CI, light pink), South (BEN_SI, dark pink), and North West (BEN_NW, purple) populations. The inset map presents the geographical locations of these populations. (*A*) Founder effects are represented as horizontal lines with widths inversely proportional to intensity. Recent population contractions with intensity inversely proportional to current population size (*t*/2*N*) are reported in white text. Population bar widths are approximately proportional to estimated population sizes. Divergence (T_DIV) and bottleneck times (T_BOT) are reported in ky (thousand years ago), assuming a mutation rate of 0.35 × 10^−8^ and 5 years per generation. Times 95% CI values are shown within brackets on the left of the time arrow. Estimated values and associated 95% CI of all parameters are reported in supplementary tables 7 and 9 in SI, Supplementary material online, and (*B*) comparison of PCA first two PC axes computed on observed and simulated data. The simulated scenario corresponds to that shown in (*A*), with parameter values taken from supplementary tables 7 and 9 in SI, Supplementary material online.

We expect recent bottlenecks to strongly dominate tiger evolutionary history. As a result, we chose the site frequency spectrum (SFS)-based methods (vs. others, e.g., GPhoCS) because they are better at detecting recent events (Beichman et al. 2018). We inferred SFS from 259,499 SNP sites in non-transcribed regions at least 50 kb away from any known gene. These were selected to minimize the effect of background selection and GC-biased gene conversion. We investigated subspecies divergence, population size changes, and gene flow. The best fit scenarios supported a very recent Holocene divergence (between 7,500 and 9,200 years ago, i.e., 1,500 and 1,840 tiger generations ago) of all tiger subspecies (fig. 3*A*) from an ancestral population. Pairwise divergence estimates based on hPSMC (Cahill et al. 2016) supported a relatively recent divergence (between 9,000 and 20,000 years ago for different subspecies pairs, supplementary fig. 13, Supplementary Material online). Demogenetic analyses supported a very strong bottleneck for the species occurring around 234,000 years ago, with most remaining lineages coalescing rapidly, which is consistent with a speciation event. This timing was consistent with signatures of population decline in the PSMC analysis (supplementary fig. 9, Supplementary Material online). The best-fit scenario, which supported divergence of the Sumatran tiger subspecies, correlates with timing of sea level rise (Heaney, 1991) and separation of the island of Sumatra. Note however that we constrained this divergence to post-date the last-glacial maximum (18,000 years ago or younger). Recent models of sea level rise suggest isolation from the mainland no later than 7,000 years ago (Bradley et al. 2016). However, to be sure that our recent divergence times did not depend on this constraint, we estimated parameters of a model without any upper bound on divergence times, which led to overall similar values for most parameters and divergence times less than 11,000 years (supplementary table 8, Supplementary Material online). Although paleohabitat reconstruction suggests the presence of a savannah corridor between the Malayan peninsula and the island of Sumatra (Bird et al. 2005), overall, our divergence estimates (between tigers in the Malayan peninsula and the island of Sumatra) are among the most recent reported for any taxa (see Leonard et al. 2015). Husson et al. (2019) suggest that divergence estimates so far may be potentially upwardly biased because they are based on mitochondrial data alone. Estimated migration rates were very low, with all populations receiving fewer than one migrant per generation; populations have been quite isolated since their initial early Holocene divergences. Additionally, we found that Sumatran and Bengal populations show evidence of a founding event, but Amur and Malayan populations do not. Both Sumatran and Amur tigers showed evidence of strong recent bottlenecks.

We further modeled the divergence within Bengal tigers into four populations: northwest India, central India, south India, and northeast India. Since PCA suggests that central and north Indian tigers are a single population and north Indian tigers were not sequenced at high coverage, we only included central Indian tigers to represent this cluster in the demographic analyses. We assessed the robustness of the northeast population being a part of the Bengal subspecies. In order to do so, the northeast population was modeled as an independent subspecies and allowed to diverge directly from the Asian metapopulation. However, such a model has a poorer fit to the data than if northeast Indian tigers are considered to be part of the Bengal subspecies (log_10_ Likelihood difference between model is 37; fig. 3*B*). Within Bengal tigers, divergences are extremely recent (within the last 2,000 years), except for the northeast tigers, which diverged early (6,800 years ago) after the separation of Bengal tigers 8,400 years ago from the ancestral Asian metapopulation. Within India, the northwest population underwent a strong bottleneck at the time of its founding. Recent bottlenecks were most severe in the northwest and south populations, whereas the northeast and central populations showed relatively weaker bottlenecks. These inferences were consistent with overall ROH for northwest and some southern individuals. Overall, tiger populations from all subspecies revealed signals of strong, recent bottlenecks, except central and northeast Bengal tigers.

The variety of analyses we conducted (model-based inference, PCA, *F*_ST_, demographic modeling) revealed that tigers from different geographic locations are genetically distinct and have been isolated from each other for as long as 8,500 to less than 2,000 years (fig. 3*A*). Genomic divergences may reflect loss of connectivity due to sea level rise, which has separated the formerly continuous Sahul subcontinent of southeast Asia into isolated islands, and changing environments due to human population size increase, including the rise of agriculture and climatic change of the mid-late Holocene. We caution that small sample sizes and assumed mutation rate may limit our ability to robustly estimate recent divergences (such as those between Bengal tiger populations).

Beichman et al. (2018) delineate five basic approaches to inferring demographic history from genomic data, which include Approximate Bayesian Computation (the most flexible), SFS-based approaches, PSMC/MSMC, IBD/IBS, and G-PhoCS and recommend SFS and ABC-based methods for inference of recent history. SFS-based methods have been used in several endangered species (expected histories of recent decline), for example, see red pandas (Hu et al. 2020) where sample sizes and effective size are comparable to our study or in ancient DNA-based human studies (e.g., Malaspinas et al. 2016). Overall, we do not expect our inferences of recent history to be biased by effective size of the species. Potential biases in the SFS due to our small sample sizes may result in fewer identified singletons, but given histories dominated by recent bottlenecks, we do not expect an SFS with a large number of singletons. Further, we conducted exploratory analyses with and without singletons, and this did not change our inferences. Finally, theoretical results suggest that accuracy of the results is affected by the number of sites, but not sample size (Terhorst and Song 2015).

In comparing our demographic history results to Liu et al. (2018), the striking difference is divergence times (estimates of effective size are comparable, we cannot compare geneflow because our model includes an “Asian metapopulation” and theirs does not). Although Liu et al. (2018) find older divergence, our results suggest recent divergence. It is possible that the differences that we observe are because of the methods used, GPhoCS is better at detecting older events, and SFS-based methods are effective for recent events. We re-iterate that our estimates of divergence time are most sensitive to assumptions of mutation rate (not effective size or sample size), but these are the same as used in Liu et al. (2018). Future research should integrate data sets and compare a variety of demographic inference methods. In the case of tigers, conducting analyses with hundreds of genomes will not be possible from wild individuals alone and will require reliance on museum specimens.

Although the timing and severity of the events differentiating tiger subspecies vary, our data and analyses confirm previous inferences (Liu et al. 2018) that the four putative subspecies of tiger are valid both geographically and genetically. The order of divergence of the subspecies from the ancestral tiger metapopulation is partially consistent with previous suggestions of tigers being isolated in Sumatra first, likely due to sea level rise (consistent in sequence but not in timing with Liu et al. [2018]), closely followed in time by those in India, then last by populations in Siberia and Malaysia (not consistent with Liu et al. [2018]).

Theoretical predictions (based on body size: Sutherland et al. [2000]) and empirical results (genetics: Joshi et al. [2013]; camera trapping: Singh et al. [2013]) suggest that individual tigers can move extraordinary distances (e.g., 300 km), even across human-dominated landscapes. Such long-range movement would result in relatively low genetic differentiation if mating between members of separate populations was frequent and successful. However, despite the possibility of long-distance dispersal, our models suggest that migration rates between tiger populations have been relatively low, emphasizing separate recent evolutionary histories, and that individual tiger movements may not represent population histories.

### Genome Scans for Selection

**Fig. 4.**
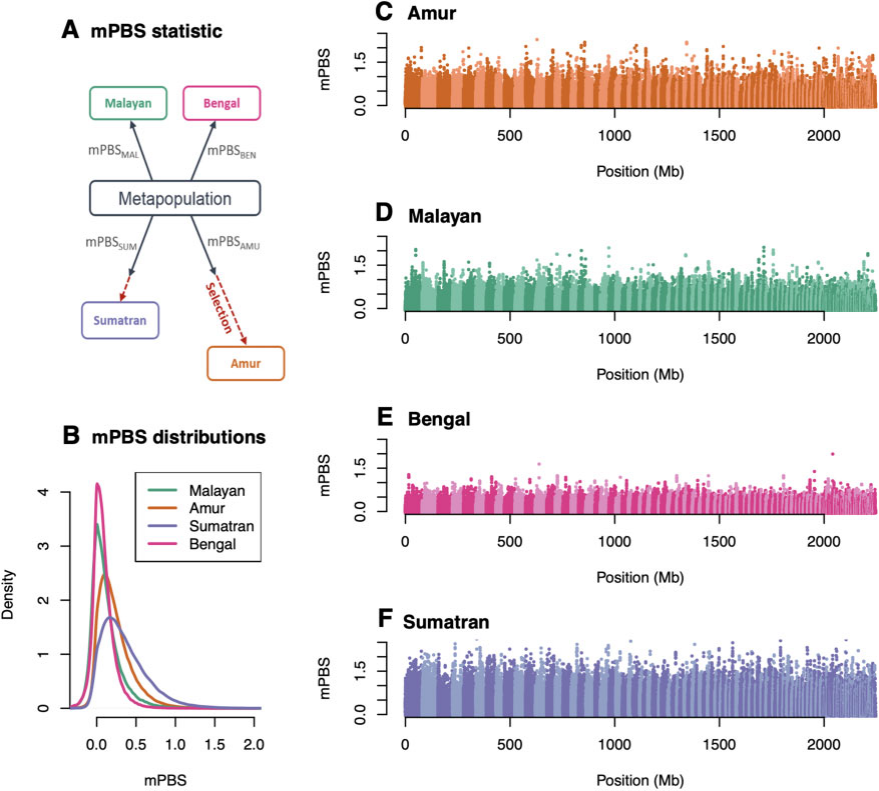
Genome scan for selection: (*A*) We present the mPBS statistic with a hypothetical model where the four populations diverge from a metapopulation, and where selection acts in both the Amur and Sumatra lineages and (*B*) the global distribution of observed mPBS for each population. Panels (*C*–*F*) correspond to the genome-wide distributions of the statistic for (*C*) Amur, (*D*) Bengal, (*E*) Malayan, and (*F*) Sumatran tigers as a function of the genomic position. Alternating light and dark colors indicate different scaffolds.

We investigated how genetic patterns might have been impacted by natural selection in the four tiger subspecies (Amur, Bengal, Malayan, and Sumatran). We computed a statistic, mPBS [metapopulation branch statistic, a simple extension of the PBS statistic of Yi et al. (2010), see Materials and Methods section], measuring the length of the branch leading to a given subspecies since its divergence from the others (fig. 4*A* and Materials and Methods section).

The genome-wide distributions of the mPBS revealed that Bengal and Malayan populations had the lowest average values, suggesting short terminal branches subsequent to the divergence of these two populations from the hypothetical metapopulation (fig. 4*B–F*). On the contrary, Amur and Sumatran tigers had high values on average (fig. 4*B–F*).

We observed little difference between transcribed and non-transcribed regions in mPBS distributions, suggesting no strong differential impact of background or positive selection in tiger coding regions (supplementary fig. 10, Supplementary Material online). Both tails of the distribution are enriched (we did not filter for mutation types), possibly caused by biased gene conversion (supplementary fig. 10, Supplementary Material online). Note that average mPBS values were higher when considering only individuals with average coverage > 10× than when comparing fewer individuals with highest coverage (supplementary fig. 10, Supplementary Material online).

Overall, the mPBS distribution obtained under the neutral demographic model (fig. 4*B*) fit very well with the observed distribution (supplementary fig. 11, Supplementary Material online), implying that most observed differences between populations could be explained by their demographic history. We predicted high mPBS values in Amur tigers and Sumatran tigers where small effective sizes would yield high levels of genetic drift, but the observed values are even higher than those expected (supplementary fig. 11, Supplementary Material online), suggesting a possible effect of natural selection on genomic diversity in these subspecies. In contrast, we observed no apparent deviation of observed mPBS values from a purely neutral model in Bengal and Malayan populations. Our ability to detect high mPBS values is contingent on the specific genomes that were used in our analyses and sample size constraints. For example, the number of Amur and Bengal tigers used in this analysis was larger than the numbers of Sumatran and Malayan tigers, which could partly explain the additional power to detect outliers. Furthermore, varying coverage can have an impact on our results. A lower average coverage in a given population will lead to an underestimation of diversity within the population. We would then overestimate *F*_ST_ and, consequently, mPBS. However, as we performed the selection analysis on the 34 samples with > 10 × average coverage only (see ^Materials and Methods section) and filtered genotypes based on depth of coverage and genotype quality, we expect to limit such biases.

Enrichment tests revealed an excess of moderately high values in Amur and Sumatran tigers rather than a few very extreme values, an observation that is compatible with the effect of polygenic selection rather than hard selective sweeps. In an attempt to identify biological functions putatively targeted by selection, we used functional enrichment tests (Daub et al. 2013; Gouy et al. 2017) based on mPBS values computed on all individuals (Amur and Sumatran) with average coverage greater than 10×. We mapped the top 0.1% regions with highest mPBS values to annotated genes (±50 kb flanking regions). One hundred and nineteen and 80 genes (in Amur and Sumatran tigers, respectively) were found within these top 0.1% regions. We identified 15 statistically significant gene ontology (GO) terms in Amur tigers and 5 in Sumatran tigers (supplementary table 10, Supplementary Material online). Out of the 15 GO categories identified in Amur tigers, 4 have an unspecific function and the 11 others are involved in lipid processing and metabolism (supplementary table 10, Supplementary Material online).

The genes responsible for the enrichment in fat metabolism-related GO terms were all included in the cellular lipid metabolic process (GO: 0044255). These included, for example, the apolipoprotein B receptor or caveolin-1 that are involved in the modulation of lipolysis. Fat metabolism enzymes included phosphatidate phosphatase (LPIN2), phospholipase B-like 1 (PLBD1), and very-long-chain (3R)-3-hydroxyacyl-CoA dehydratase 2. We also identified genes involved in the mitochondrial respiratory chain: a cytochrome P450 subunit (*CYP1A2*) and the mitochondrial lipoyl synthase. Cardiolipin synthase is involved in the synthesis of cardiolipin, an important phospholipid of the mitochondrial membrane critical to mitochondrial function. Finally, thromboxane-A synthase is involved in vasoconstriction and blood pressure regulation.

In Sumatran tigers, significant GO terms were related to cell development regulation: regulation of neuron projection development (GO: 0010975), regulation of anatomical structure size (GO: 0090066), and regulation of cell development (GO: 0060284). These four terms contain the same six genes: tyrosine-protein kinase (*RYK*), E3 ubiquitin-protein ligase (*RNF6*), low-density lipoprotein receptor-related protein 1 (*LRP1*), angiotensin-converting enzyme (*ACE*), Rap1 GTPase-activating protein 2 (*RAP1GAP2*), and B2 bradykinin receptor (*BDKRB2*). These genes are involved in morphological development, and selection targeting these loci may help to explain why Sumatran tigers are in general smaller than other subspecies. Two other terms related to toxic substance processing, are response to toxic substance (GO: 0009636) and organophosphate biosynthetic process (GO: 0090407).

### What Evolutionary Processes Dominate the Evolution of Tigers and Their Subspecies?

Our models and analyses suggested relatively recent divergence between tiger populations (a maximum of 20,000 or so years vs. 68,000 years inferred by Liu et al. [2018]), highlighting the role of drift/stochastic processes in recent tiger evolution. Our inference is contingent on a mutation rate of 3.5 × 10^−8^ from Liu et al. (2018). Discrepancy between our and Liu et al. (2018) estimates could also be due to differences in filtering criteria (Liu et al. [2018] use min DP = 4, min GQ = 20, whereas we have used minDP = 10, and min GQ = 30) or the sites considered for the analyses. We used a restricted set of sites that were far from coding regions and thus minimally affected by background selection and biased gene conversion (about 100 Mb worth of data), whereas Liu et al. (2018) used about 44 Mb worth of data (for GPhoCS analyses), background selection, or biased gene conversion. Our results consistently underline the genome-wide importance of genetic drift. Despite recent divergence, we found significant genetic differentiation between tiger populations, possibly because of the intense bottlenecks these populations have experienced.

Our results suggested that Amur tiger genomes demonstrate signals of selection, with possible adaptations to colder environments. We do not think that the signatures of selection we identify in Amur tigers (while Liu et al. [2018] did not) is due to our larger sample sizes. Genes and pathways involved in lipid metabolism are under selection in two human populations that live in cold environments, including Greenlandic Inuit (Fumagalli et al. 2015) and Indigenous Siberians (Hallmark et al. 2018). Polar bear genomes also reveal signatures of selection on lipid metabolism genes (Liu et al. 2014). Understanding the distribution of adaptive variants could be important for future conservation efforts, especially if priority was placed on preserving these cold-adapted populations, which may be disadvantaged under future warming scenarios.

Sumatran tigers appear to have experienced strong genetic drift following vicariance from mainland southeast Asia, maintained a smaller effective population size, and have experienced a strong recent bottleneck. Although Liu et al. (2018) suggested that selection for body size targeted the *ADH7* gene, we did not detect any signature of selection at this locus in our Sumatran samples. However, we identify alternative candidate genes that are potentially involved in body size, such as the genes found in the *Regulation of anatomical structure size* GO term (supplementary table 10, Supplementary Material online). We caution that it is difficult to truly distinguish among all population genetic processes, especially selection, without more data, and assignments of GO categories designed from model organisms are only a substitute for more definitive tests of selection. Differences between our study and Liu et al. (2018) may be due to the improved quality of the genome we built and mapped to, which generally increases the accuracy of gene finding and annotation software. Alternatively, Liu et al. (2018) had larger sample sizes for Sumatran tigers, and the differences could be due to the use of different data sets. However, the locus identified as under selection by Liu et al. (2018) had normal levels of variation in our Sumatran tiger genomes. Careful sampling of known origin wild individuals, high coverage sequencing, and synthetic analyses will be critical to resolve these differences.

We did not detect signatures of selection or extensive gene flow into Malayan and Bengal tiger genomes, suggesting that their genomic variation was due primarily to drift. Figure 2 (see supplementary tables 7 and 9 for magnitude of recent bottleneck) suggests that at least some Indian tigers have experienced intense founder events (e.g., BEN_NW), intense recent bottlenecks and population structuring, and mPBS, and figure 4 substantiates a relatively stronger role of drift (compared with Malayan tigers) in shaping genome-wide variation.

### Conservation Implications

With different individuals and much larger samples size for several subspecies, we show that tigers (from the four sampled subspecies) have recently differentiated through contrasting histories of drift and selection, making each subspecies evolutionarily unique. For Amur tigers, our results from population structure analyses, demographic history-based divergence, and signatures of possible selection reaffirm their unique management status [as suggested by Liu et al. (2018) and Wilting et al. (2015)]. Increasing population size and enabling gene flow over the long term might augment the currently low genetic diversity in this population. For Bengal tigers, recent fragmentation and ensuing loss of connectivity appear to result in significant autozygosity. Restoring and maintaining gene flow between populations through habitat corridors may be more important (along with increasing population numbers) here. Assisted geneflow could be considered as a management strategy, especially when inbreeding is associated with loss of fitness and potentially inbreeding depression. Within Bengal tigers, we suggest that the management status of northeast Indian tigers be re-evaluated given their antiquity and potential genetic distinctiveness (Kolipakam et al. 2019). The surprisingly high (relative) genetic variation and population differentiation in Malayan tigers bodes well for their future survival. It will be critical for future conservation efforts to prioritize population recovery and gene flow through connectivity and to promote population size increases. Critical to such action is a better understanding of within population genetic variation using spatially explicit, non-invasive sampling (e.g., Khan et al. 2020). Finally, Sumatran tigers should be managed separately because like Liu et al. (2018) and Wilting et al. (2015), our results re-iterate their uniqueness. Their genomes show signatures of selection for genes regulating body size [broadly consistent with the findings in Liu et al (2018)].

In summary, ongoing human impacts like fragmentation will likely continue to disrupt natural evolutionary processes in wild tigers. Managing local populations to minimize human impacts maybe the key to species survival and the important conservation strategy for the anthropocene. Additional historical and genomic sampling may provide an informed roadmap for genetic rescue and augmentation. Considering the contrasting results found between our study and those from Liu et al (2018), it is also a reminder that we need to carefully and critically interpret the results from genomics analyses for endangered species with limited sample sizes, especially when they could impact management decisions. It is clear that not all the questions regarding tiger evolution have been definitively answered despite two wide-range sampling efforts. Ongoing method development and increased collaboration will help gain better insights into the evolutionary history of species of conservation concern and better advice for their futures.

## Materials and Methods

### Sample Collection

We obtained tissue, blood, or serum samples from as many geographically distinct tiger populations as possible. This amounted to 65 samples from four tiger subspecies including 21 Bengal tigers (*P. t. tigris*), 19 Amur tigers (*P. t. altaica*), 15 Malayan tigers (*P. t. jacksoni*), and 11 Sumatran tigers (*P. t. sumatrae*). A final list of samples sequenced, and their sources are available in supplementary table 1, Supplementary Material online. We also included one already sequenced sample, which brought the sample total to 66 (see supplementary table 1, Supplementary Material online).

### Reference Assembly Sequencing and de Novo Assembly

In order to better understand genome-wide variation and call variants reliably, we first built a new tiger genome assembly using the 10× Genomics Chromium Platform for a wild-caught Malayan individual. We received whole blood from a wild born Malayan tiger (*P. t. jacksoni*) sampled by the El Paso zoo, Texas on 7/28/2016, collected as part of a routine health check-up. We immediately froze the sample at −80°C until it was shipped on dry ice to the Barsh lab at HudsonAlpha for extraction and delivery to the Genome Services Lab (GSL) at HudsonAlpha Institute for Biotechnology, Huntsville, Alabama. DNA was extracted and purified using the Qiagen MagAttract HMW DNA kit. GSL staff prepared a linked-read sequencing library using the Chromium controller. The library was sequenced on one lane of a HiSeqX. We assembled the genome using the SuperNova assembly software (1.1.4) provided by 10× Genomics using the standard pipeline. We refer to this assembly as Maltig1.0 hereafter.

### Whole Genome Resequencing and Variant Discovery

Details on DNA extraction, library preparation, and variant discovery methods can be found in Supplementary Material online.

### Population Structure

We first investigated admixture and structure between populations using Plink2 (Chang et al. 2015). We used VCFtools to filter the initial variant call file using “max-missing 0.95” and “maf 0.025” and removed sites with missing data and rare variant calls. We then converted to Plink’s “.ped/.map” format using VCFtools and subsequently converted to “.bed/.bim/.fam” format within Plink2 using the flag “–make-bed.” PCA was then run on the resulting bed file using the flag “–pca 10” that computed the variance-standardized relationship matrix. PCAs were then plotted using R. For smaller runs, an additional step was added within Plink2 to first calculate the frequencies using the flag “–freq.” Subsequently, PCA was run using the “–pca” flag and inputting the frequency file using the “–read-freq” flag. We used this protocol on the vcf with all individuals, and subsequently, we divided the vcf into the putative subspecies for within subspecies runs.

The program ADMIXTURE was used to infer structure between populations and inform clusters that represent populations with distinct histories (Alexander et al. 2009). ADMIXTURE uses maximum likelihood-based models to infer underlying ancestry for unrelated individuals. We used the filtered data set (VCFtools max-missingness cutoff of 95%, minor allele frequency cutoff of 0.025) and resulting Plink formatted files for input into the software. In order to infer the most likely value of *K*, values of 2–8 were run. We also performed *K* validation in order to compute the cross-validation error for each value of *K*, by using the “–cv” flag within the program. The value with the least error is informative of the best value of *K* for the data.

### Rarefaction Analysis

To ensure that our data were reflective of the diversity within each subspecies/unit as defined by ADMIXTURE, we used the program ADZE (Szpiech et al. 2008). ADZE runs a rarefaction analyses on polymorphism data in order to estimate the number of alleles private to any given population (not found in any other population), considering equal-sized subsamples from each input population. In addition, the program calculates distinct alleles within each population. We calculated the private alleles across the four main populations/sub-species as designated by the ADMXITURE software, in addition to the distinct alleles within each of the four populations individually.

### Population Differentiation and Diversity

We calculated pairwise *F*_ST_ between each subspecies group as defined by ADMIXTURE using VCFtools. Variant call data were subdivided into sub-species based on PCA (Bengal, Sumatran, Amur, and Malayan as subgroups) and were used to compute pairwise *F*_ST_ between each group. Nucleotide diversity (pi) was calculated using VCFtools.

In order to detect the number of single nucleotide variants, the data were filtered using VCFtools (Danecek et al. 2011) to a minimum base quality of 30, genotype quality (GQ) of 30, and depth of 10. Additionally, we filtered for minor allele frequency of 0.025 and allowed a maximum 5% missing data in any loci. RTG tools (https://www.realtimegenomics.com/products/rtg-tools) vcfstats were used to calculate the total number of heterozygous SNP sites for each individual. These values were then plotted alongside comparable estimates for other species reported in Abascal et al. (2016).

### Ancient Demographic History

PSMC (Li and Durbin, 2011) is a single genome method to detect historical effective population size. In order to estimate historical population size changes for the different subspecies, we removed sex chromosome scaffolds for AMU1, MAL1, SUM2, and BEN_SI3 (the highest coverage individual for each subspecies). The procedures for sex chromosome filtering can be found in the supplementary text (and supplementary fig. 1, Supplementary Material online). Additionally, sites with a minimum of half the average sequencing depth or twice the average sequencing depth were filtered out while calling variant sites. The resulting scaffolds were then used to estimate the effective population size across 34 time intervals as described in Li and Durbin (2011). One hundred rounds of bootstrap replicates were performed.

### Runs of Homozygosity

To estimate ROH, we used the filtered SNPs from the autosomal scaffolds. Individuals with more than 10× average coverage were grouped as per subspecies. We used BCFtools/RoH (Narasimhan et al. 2016) to estimate ROH. The autozygous runs obtained were classified into various lengths (runs between 10 and 100 kb, runs between 100 kb and 1 Mb, and runs longer than 1 Mb). Proportion of genome in ROH for 1 Mb was estimated as the total length of the genome in more than 1 Mb runs divided by the total length of autosomal scaffolds. Similar calculations were made for 100 kb to 1 Mb runs and for 10–100 kb runs except the length of the genome longer than 1 Mb and 100 kb were subtracted from total length of autosomes, respectively. We used an additional sliding window approach, details of which can be found in the supplementary methods.

### Demographic History with SFS and Coalescent Models

#### Demographic Models

Data filtering procedures for the demographic models can be found in the supplementary text. Using the program fastsimcoal 2 (Excoffier et al. 2013), we performed demographic estimations of the model shown in figure 3*A* on two data sets in two consecutive steps such as to reduce the number of parameters to estimate simultaneously. The first step consisted in estimating the demography (24 parameters) of four tiger subspecies (Malaysia—MAL, Sumatra—SUM, Bengal—BEN, and Amur—AMU) using the individuals of each subspecies that had the highest coverage. We thus selected three SUM individuals, three BEN individuals from South India (BEN_SI), four MAL individuals, and three AMU individuals, which all had >20× coverage on average (see list in supplementary table 3, Supplementary Material online). We modeled the four-subspecies as belonging to a large Asian metapopulation, from which they would have diverged some time ago while still receiving some continuous gene flow from the metapopulation. Note that this continent–island population structure amount to modeling a set of populations having gone through a range expansion (Excoffier 2004). We assumed that each of the four subspecies could have gone through two distinct bottlenecks, one that would have occurred at the time of the separation from the Asian metapopulation to mimic some initial founder effect and one that would be recent to mimic habitat deterioration. We also assumed that the Asian metapopulation could have gone through an ancestral bottleneck sometime in the past.

The second step used estimated parameters in a more complex model including the specific demography of four Bengal tiger populations (24 new additional parameters). To this aim, as in the previous analysis, we selected individuals with the highest coverage (>20×) from each population (see supplementary table 1, Supplementary Material online, samples used represented in supplementary table 3, Supplementary Material online). No individuals from BEN_NOR were included as their coverage was low, and they are part of the same genetic cluster as BEN_CI. To correctly estimate the relationship between these populations and the other subspecies, we also included three MAL individuals in this analysis. The new model included all the parameters from the previous model, fixed at their previously estimated values, except some parameters re-estimated for the BEN_SI population, which was now assumed to have diverged from an Indian metapopulation at some time in the past, like the other three BEN tiger populations. We also estimated the size and the divergence of the BEN metapopulation from the Asian metapopulation. We allowed the sampled BEN populations to have gone through two bottlenecks (an initial founder effect and a recent bottleneck). The parameters estimated in these two steps are shown in supplementary tables 7 and 9, Supplementary Material online, and the resulting demography is sketched in figure 3. Details of parameter estimation are in the supplementary method, Supplementary Material online.

#### Genome Scan for Selection

To detect the footprints of natural selection in different tiger subspecies, we analyzed individuals with coverage > 10× from four subspecies (*n* = 34). We filtered out genotypes with depth of coverage < 10 (DP) and GQ < 30]. We excluded scaffolds shorter than 1 Mb. We kept sites with no missing data among the 34 individuals.

We considered the four subspecies as four populations and computed pairwise *F*_ST_ values along the genome over 50-kb sliding windows (with a step of 10 kb) using the R package PopGenome (Pfeifer et al. 2014). *F*_ST_ are computed with the estimator described in Hudson et al. (1992). We then computed a measure of selection similar to the PBS (Yi et al. 2010). The PBS statistic is based on a three-population comparison and measures the length of the branch leading to a given population since its divergence from the two other populations. This statistic is not able to accommodate more than three populations and relies on a tree-based model that does not correspond to tigers’ demographic history. Therefore, we extended this statistic to the case of four populations under a more suitable model than a tree-based one. Furthermore, using all four populations allows to better characterize the differences that are exclusive to specific branch. We define:

$${\mathrm{mPBS}}_{a}=\frac{2\left(T_{\mathrm{ab}}+T_{\mathrm{ac}}+T_{\mathrm{ad}}\right)-(T_{\mathrm{bc}}+T_{\mathrm{bd}}+T_{\mathrm{cd}})}{6},$$

where$\;T_{ij}$ is the divergence time, in generations, between population *i* and *j* (Nei 1972):

$$T_{ij}=-\log\left(1-F_{\mathrm{ST}}^{ij}\right).$$

This statistic assumes that each population diverged from a metapopulation independently and that no migration occurred following divergence. It measures the length of the branch leading to a given lineage since its divergence from the metapopulation. Selection in a given lineage will lead to a much longer terminal branch than under neutrality. This would translate to extreme mPBS values.

To compare observed mPBS values to expectations under the tigers’ demographic history, we simulated one million genomic windows using the demographic model inferred previously. Window size and sample size for each population are the same as in the observed data set. Parameter values are fixed and correspond to the maximum likelihood estimates (supplementary tables 7 and 9, Supplementary Material online). Then, we computed the mPBS statistic for each population to generate a null distribution. Observed and simulated distributions were then represented to see whether observed values deviated from neutral expectations.

Enrichment tests were used to detect the targets of selection. These tests are a conservative approach to detect selection because they are less susceptible to the influence of non-selective forces. To identify putative genes under selection, we considered predicted genic regions of the tiger genome for which a homolog has been annotated using Exonerate (protein2genome model). To avoid spurious enrichment signals due to the presence of multiple homologs for a single gene, we kept only one homolog for each predicted gene. If different homologs on the same strand overlap, we pick the first one and ignore the others. We retained a total of 12,771 genes after filtering.

We also checked whether some GO terms (Ashburner et al. 2000; Mi et al. 2016) were enriched across candidate genes (Fisher’s exact test performed on human GO terms). Genes (±50 kilobases flanking regions) were considered as candidates if they overlapped with a window that was in the top 0.1% of mPBS value of a given population. The reference list of genes for the enrichment test is set as the list of genes after filtering (12,771 genes).

## Supplementary Material

msab032_Supplementary_Data
