# Supplementary material for: Recent Evolutionary History of Tigers Highlights Contrasting Roles of Genetic Drift and Selection
Source: Mol Biol Evol. 2021 Feb 16;38(6):2366–79. doi: 10.1093/molbev/msab032 (PMC8136513; doi:10.1093/molbev/msab032)
Supplement: msab032_Supplementary_Data [file msab032_supplementary_data.docx]

**Supplementary methods**

**DNA Extraction and Library Preparation**

We extracted DNA from samples using the Qiagen DNeasy blood and tissue kits (Catalogue #69504) and quantified DNA concentrations with the Qubit dsDNA HS assay kit (Q32851). As a number of our samples yielded very low amounts of DNA (< 1 ng), we used an approach that scales down the input reagents and necessary DNA input and modified the Illumina Nextera kit protocol (Baym et al. 2015). Khan et al. (2020) have shown that such methods work well for very low DNA concentrations for tigers. Genomic libraries were run on a 2100 BioAnalyzer (Agilent Technologies using High Sensitivity DNA Chips (Catalog #5067-4626) to determine the quality, quantity, and fragment size distributions. Libraries were sequenced on Illumina HiSeqX, HiSeq 4000, and HiSeq 2500 for between 5-25x coverage using paired-end 2x150bp reads (Supplementary Table 1).

***De novo* genome quality assessment and annotation**

We identified repetitive regions in the genomes in order to perform repeat analysis and to prepare the genomes for annotation. Repeat annotation was accomplished using  homology-based and *ab-initio* prediction approaches. We used the felid RepBase (<http://www.girinst.org/repbase/>; Jurka et al. 2005) repeat database for the homology-based annotation within RepeatMasker (http://www.repeatmasker.org; Smit et al. 1996). RepeatMasker setting -gccalc was used to infer GC content for each contig separately to improve the repeat annotation. We subsequently performed *ab-initio* repeat finding using RepeatModeler (<http://repeatmasker.org/RepeatModeler.html>; Smit and Hubley 2008). RepeatModeler does not require previously assembled repeat databases and identifies repeats in the genome using statistical models.

We performed gene annotation with the Maker3 annotation pipeline using protein homology evidence from the felid, human, and mouse UniProt databases. Gene prediction was performed with the Augustus Human model. We assessed continuity and genome quality using the Assemblathon2 scripts in addition to BUSCOv4 (Simão et al. 2015; Waterhouse et al. 2018) using the mammaliaodb10 lineage dataset, which contains 9,226 BUSCO groups.

**Variant Discovery**

We trimmed reads prior to mapping with TrimGalore (Krueger 2015), then mapped reads to the Maltig1.0 reference genome using BWA-MEM (Li 2013) and sorted and indexed using SAMtools (Li et al. 2009). We marked duplicate reads with the Picard Tools `MarkDuplicates` command (<http://broadinstitute.github.io/picard>). We then called variants from the resulting BAM files using FreeBayes (Garrison & Marth 2012). The resulting VCF file was filtered with VCFtools (Danecek et al. 2011) to a minimum quality of 30, a genotype quality of 30, maximum of 2 alleles, Hardy-Weingburg p-value of 0.0001, minimum allele frequency of 0.01, and a minimum minor allele count of 3. The pipeline was managed and parallelized using NextFlow (Tommaso et al. 2017).

**Sex chromosome filtering for PSMC analysis**

We excluded the sex chromosomes for demographic analyses because their effective population size differs from that of autosomes. We identified potential sex chromosome scaffolds using three strategies: (a) The assembled tiger scaffolds were aligned to the domestic cat genome assembly (Felis_catus_9.0) using BLAST (Altschul et al. 1990). The top hits for each alignment were chosen. All tiger scaffolds that aligned with the domestic cat X chromosome scaffolds were marked as a sex chromosome; (b) The re-sequencing reads for MAL1 (also the individual used for 10x assembly) were aligned to the tiger genome assembly. All the scaffolds sequenced at half the average depth or twice the average depth were marked as sex chromosome scaffolds; (c) Since the sex for many tigers in this dataset are known, a statistical association between scaffolds at half the average sequencing depth and zero sequencing depth was established (Supplementary method Figure 1). Females had few scaffolds sequenced at zero depth, and these represent Y chromosomes. Males had certain scaffolds sequenced at half the average depth, representing potential X chromosome. Using a conservative approach, all the scaffolds identified in even one of these analyses were marked as sex chromosomes and removed.

**Supplementary Figure 1**: Association between sex and coverage of various scaffolds. Coverage is normalized per individual.

**Demographic history with hPSMC**

We used the default methods described in <https://github.com/jacahill/hPSMC> and discussed in Cahill et al. (2016) for estimating pairwise divergence times between populations. Briefly, we created consensus fasta files of haploid genomes from sex scaffold filtered BAM files used in PSMC. We then created pseudo-diploid genomes consisting of genomes of one individual each from two populations in a pairwise manner. These pseudo-diploid genomes were divided into sequence bins of 10bp each. Coalescent times were estimated for each bin and plotted as done for PSMC (Li & Durbin 2011).

*Parameter estimation and fastsimcoal2 command line*

*Fastsimcoal2* (Excoffier et al. 2013) was used to estimate parameters from the multidimensional site frequency spectrum (SFS) computed on non-coding regions at least 50 Kb away from known genes. The multidimensional SFS was computed with the program Arlequin ver 3.5.2.2 (<http://cmpg.unibe.ch/software/arlequin35>) on polymorphic sites matching the filtering criteria listed above. In order to infer absolute values of the parameters, we used a mutation rate of 0.35e-8 per site per generation estimated in a previous paper on tiger demography (Liu et al. 2018), and we assumed that the proportion of monomorphic sites passing our filtering criteria in each 1Mb segment was identical to the fraction observed in polymorphic sites. Parameter estimation was obtained by maximum-likelihood estimation obtained from 100 independent runs of *fastsimcoal2*, 60 expectation conditional maximization (ECM) cycles per run and 500 thousand coalescent simulations per estimation of expected SFS. The *fastsimcoal2* command line used for the estimation was of the type

fsc -t xxx.tpl -n500000 -d -e xxx.est -M -l30 -L60 -q -C1 --multiSFS -c1 -B1

where fsc is the *fastsimcoal2* program and xxx the generic name of the input files. The .est and .tpl input files used for inference on the two datasets are listed in the Supplementary material.

Confidence intervals were estimated via a block bootstrap approach. We generated 100 bootstrapped SFS by resampling (and adding) the SFS from 1 Mb segments of concatenated non-coding segments along the genome, and for each of these bootstrapped samples we re-estimated the parameters of the model using 10 *fastsimcoal2* independent runs starting at the maximum-likelihood parameter values. Again, we used 60 ECM cycles for each run and performed 500,000 simulations for estimating the expected SFS under a given set of parameter values. The limits of 95% confidence intervals were estimated by computing the 2.5% and 97.5% quantiles of the distribution of 100 maximum-likelihood parameter values.

**Estimation of ROH using sliding window approach**

Within individual genomes, we identified ROH using the methods first implemented in Pemberton et al. (2012). For each of the four tiger populations, we estimated the allele frequencies at each SNP using the observed allele frequencies for the four populations in our data set. To identify ROH, we employed a likelihood method adapted by Pemberton et al. (2012). This approach, which forms the basis of the ROH inference program GARLIC (Szpiech et al. 2017), considers a sliding window of *n* SNPs that moves along the chromosome in single SNP increments. To ensure robustness in our results, we repeated the ROH identification procedure with three values of *n*: 100, 250, and 700. Within each window, a log-likelihood score was computed for each SNP, comparing the hypothesis that the SNP is autozygous to the hypothesis that it is non-autozygous, allowing for an error term that accounts for mutation or genotyping error. As in Pemberton et al. (2012), we set the error parameter to 0.001. The overall score of a window is then the sum of the scores of the SNPs it contains, with an observed homozygous SNP contributing a positive score (unless the SNP is monoallelic in the population in question), and an observed heterozygous SNP contributing a negative score.

Following that, all windows with an overall score of 0 or greater were taken to be in an ROH, with overlapping windows merged and considered as part of a single ROH. The bp length of each ROH is taken to be the length of the interval between the its two most extreme SNPs, including the endpoints. For the three values of *n*, we then plotted the total length of ROH present in each individual, as well as the total length of long ROH (≥ 1Mb) present in each individual (Supplementary Figure 8).

**Demographic history with SFS and coalescent models**

*Data filtering*

In order to minimize the effect of background selection (BGS) that could bias demographic inferences (Pouyet et al. 2018), we excluded genic regions and those within 50kb of gene transcripts. We concatenated the remaining sequences in a single fictive chromosome and divided it into smaller segments of 1Mb in length. These 1Mb segments were used for block-bootstrap resampling for the estimation of parameter confidence intervals (see below).

From the VCF including information on polymorphic sites, we used the Snow leopard genomic sequence (Cho et al. 2013) as a reference to filter out triallelic sites and those sites for which the Snow leopard variant was missing. We further excluded sites where any individual had a site with a coverage (DP) lower than 10 or a genotype quality (GQ) lower than 30. To exclude the effect of GC-Biased Gene Conversion (BGC) on demographic inference, (Pouyet et al. 2018), we only considered sites with A ↔ T or C ↔ G mutations that are not affected by BGC. Finally, for any given data set defined below, sites with any missing data for one of the filtering step described above were also excluded. The number of monomorphic sites passing all our filtering criteria including BGC was computed by assuming that BGC-free monomorphic sites would have a similar frequency among all monomorphic sites than BGC-free polymorphic sites among all polymorphic sites.

**References**

1. Altschul SF, Gish W, Miller W, Myers EW, Lipman DJ. 1990. Basic local alignment search tool. *Journal of molecular biology*, *215*(3), pp.403-410.
2. Baym M, Kryazhimskiy S, Lieberman TD, Chung H, Desai MM, Kishony R. 2015. Inexpensive multiplexed library preparation for megabase-sized genomes. *PloS one*, *10*(5), p.e0128036.
3. Bradnam KR, Fass JN, Alexandrov A, Baranay P, Bechner M, Birol I, Boisvert S, Chapman JA, Chapuis G, Chikhi R, et al. 2013. Assemblathon 2: evaluating de novo methods of genome assembly in three vertebrate species. *GigaScience*, *2*(1), pp.2047-217X.
4. Cho YS, Hu L, Hou H, Lee H, Xu J, Kwon S, Oh S, Kim HM, Jho S et al. 2013. The tiger genome and comparative analysis with lion and snow leopard genomes. *Nature communications*, *4*, p.2433.
5. Di Tommaso P, Chatzou M, Floden EW, Barja PP, Palumbo E, Notredame C. 2017. Nextflow enables reproducible computational workflows. *Nature biotechnology*, *35*(4), pp.316-319.
6. Garrison E, Marth G. 2012. Haplotype-based variant detection from short-read sequencing. *arXiv preprint arXiv:1207.3907*.
7. Jurka J, Kapitonov VV, Pavlicek A, Klonowski P, Kohany O, Walichiewicz J. 2005. Repbase Update, a database of eukaryotic repetitive elements. *Cytogenetic and genome research*, *110*(1-4), pp.462-467.
8. Khan, A., Patel, K., Bhattacharjee, S., Sharma, S., Chugani, A.N., Sivaraman, K., Hosawad, V., Sahu, Y.K., Reddy, G.V. and Ramakrishnan, U., 2020. Are shed hair genomes the most effective noninvasive resource for estimating relationships in the wild?. *Ecology and Evolution*.
9. Krueger F. 2015. Trim galore. *A wrapper tool around Cutadapt and FastQC to consistently apply quality and adapter trimming to FastQ files*, *516*, p.517.
10. Li, H., 2013. Aligning sequence reads, clone sequences and assembly contigs with BWA-MEM. *arXiv preprint arXiv:1303.3997*.
11. Liu YC, Sun X, Driscoll C, Miquelle DG, Xu X, Martelli P, Uphyrkina O, Smith JL, O’Brien SJ, et al. 2018. Genome-wide evolutionary analysis of natural history and adaptation in the world’s tigers. *Current Biology*, *28*(23), pp.3840-3849.
12. Pemberton TJ, Absher D, Feldman MW, Myers RM, Rosenberg NA, Li JZ. 2012. Genomic patterns of homozygosity in worldwide human populations. *The American Journal of Human Genetics*, *91*(2), pp.275-292.
13. Pouyet F, Aeschbacher S, Thiéry A, Excoffier L. 2018. Background selection and biased gene conversion affect more than 95% of the human genome and bias demographic inferences. *Elife*, *7*, p.e36317.
14. Smit AF, Hubley R, Green P. 1996. RepeatMasker.
15. Smit AF, Hubley R. 2008. RepeatModeler Open-1.0. *Available fom http://www. repeatmasker. org*
16. Szpiech ZA, Jakobsson M, Rosenberg NA. 2008. ADZE: a rarefaction approach for counting alleles private to combinations of populations. *Bioinformatics*, *24*(21), pp.2498-2504.
17. Simão FA, Waterhouse RM, Ioannidis P, Kriventseva EV, Zdobnov EM. 2015. BUSCO: assessing genome assembly and annotation completeness with single-copy orthologs. *Bioinformatics*, *31*(19), pp.3210-3212.
18. Waterhouse RM, Seppey M, Simão FA, Manni M, Ioannidis P, Klioutchnikov G, Kriventseva EV, Zdobnov EM. 2018. BUSCO applications from quality assessments to gene prediction and phylogenomics. *Molecular biology and evolution*, *35*(3), pp.543-548.

**Other supplementary figures**


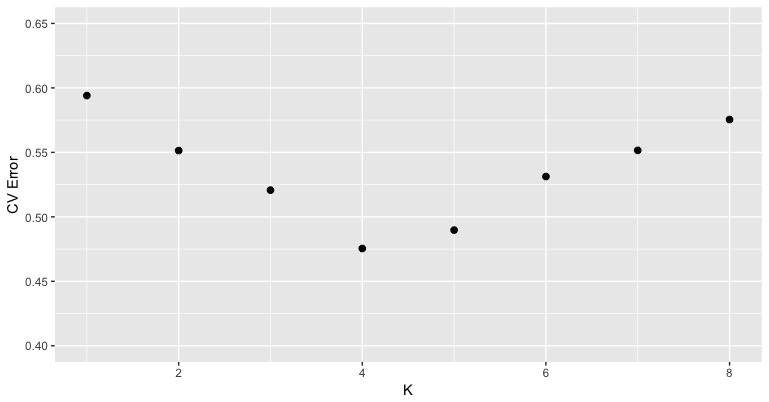


**Supplementary Figure 2**: Cross-validation error for runs of ADMIXTURE across different values of K.

**Supplementary Figure 3**: ADMIXTURE plots for all tigers for K values 2-9.


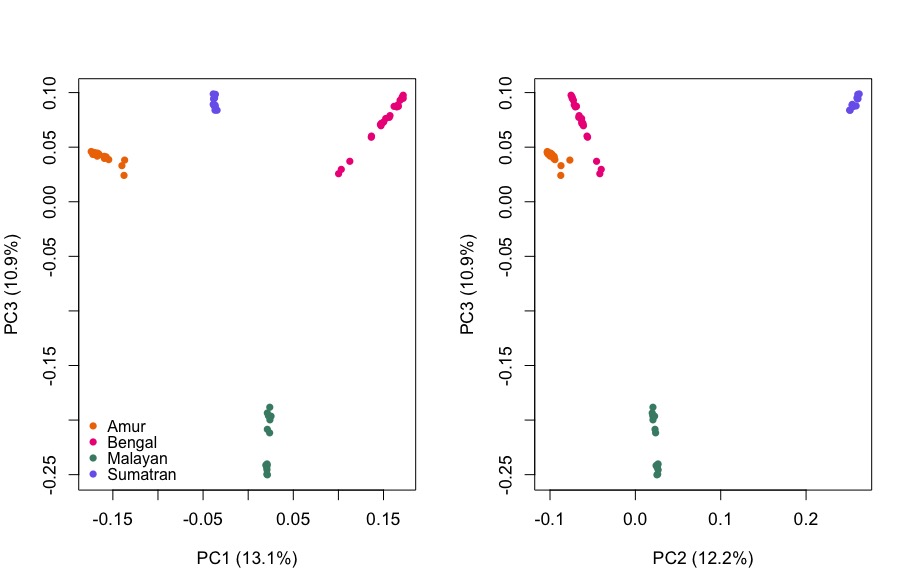


**Supplementary Figure 4**: Additional PCs (1-3) for all tigers as computed by PLINK2.


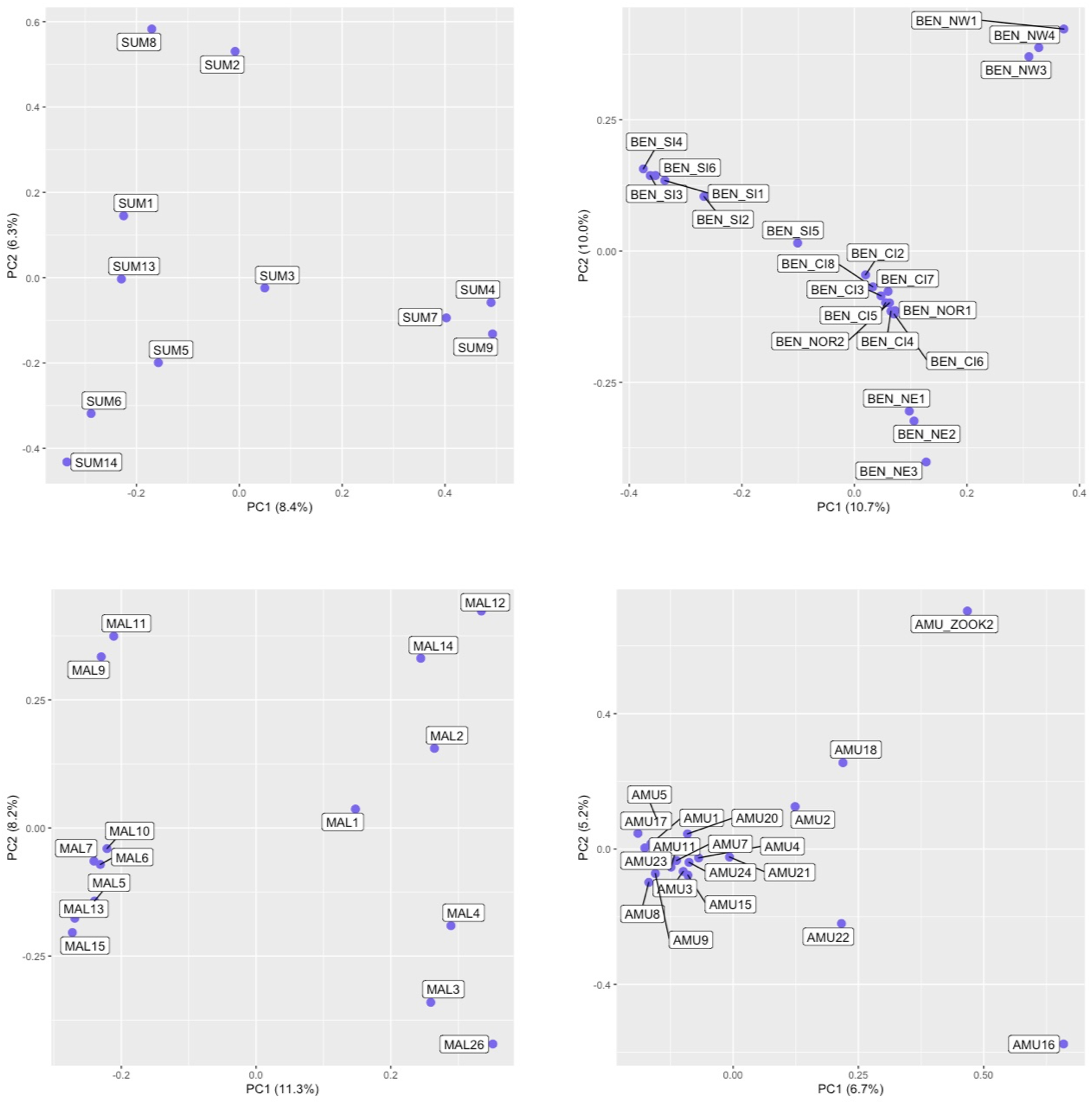


**Supplementary Figure 5 a, b, c, d**: Within subpopulation PCA for Sumatran (top left), Bengal (top right), Malayan (bottom left), Amur (bottom right), and Sumatran populations as computed by VCFtools with individuals labeled.


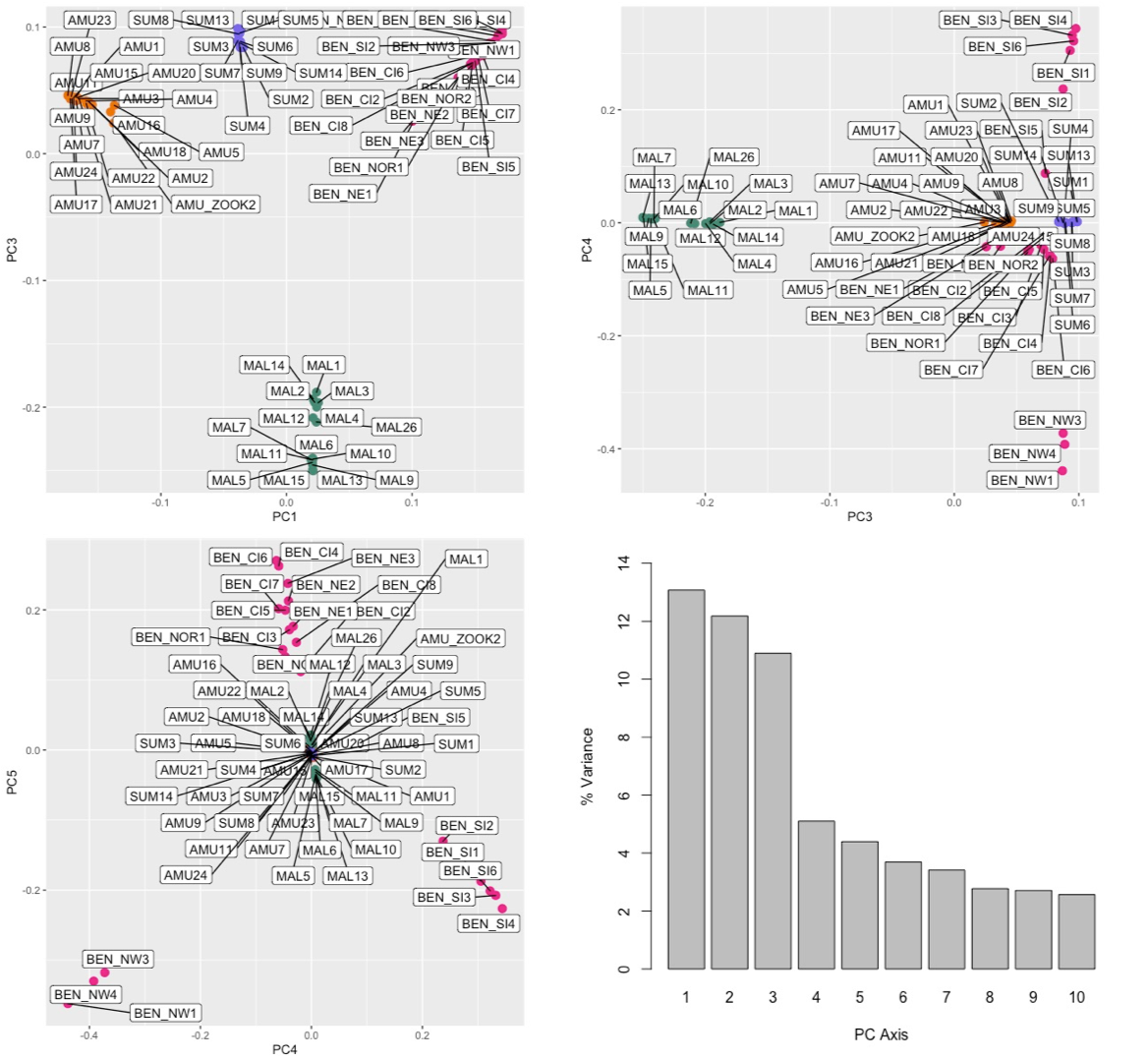


**Supplementary Figure 6**: PCA separation of individuals across (top left) PC axis 1 vs 3 and (top right) PC axis 3 vs 4 (bottom left) PC axis 4 vs 5, and (bottom right) the variance explained across the first 10 PC axes.

**Supplementary Figure 7**: Encounter rate for heterozygous SNVs as across the genome for various species. For most species, these are based on published estimates from single genomes. The error bar for the four tiger subspecies represented standard deviation across the mean.

**Supplementary Figure 8**: Runs of Homozygosity predicted by GARLIC (Szpiech et al. 2017).

**Supplementary Figure 9**: PSMC plot for a single high coverage individual from each subspecies. Our plot is consistent with that observed by Liu et al (2018).


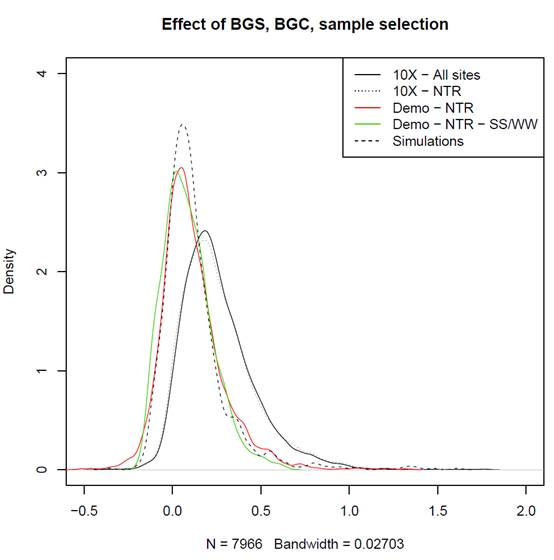


**Supplementary Figure 10**: Effect of background selection, biased gene conversion and sample selection on mPBS distributions in Siberian tigers. The black solid line represents mPBS values obtained when considering all sites and all individuals with average coverage above 10X (“10X – All sites”). The dashed thin black line corresponds to the same individuals but excluding transcribed regions and 50 kb flanking regions (“10X – NTR”). The red curve is computed using the same individuals that were used for demographic inference in non-transcribed regions (“Demo – NTR”). The green curve is obtained after filtering out sites potentially affected by biased gene conversion (“Demo – NTR – SS/WW”). Finally, the dashed black line corresponds to simulated values under the demographic model inferred from NTR-SS/WW sites (“Simulations”).


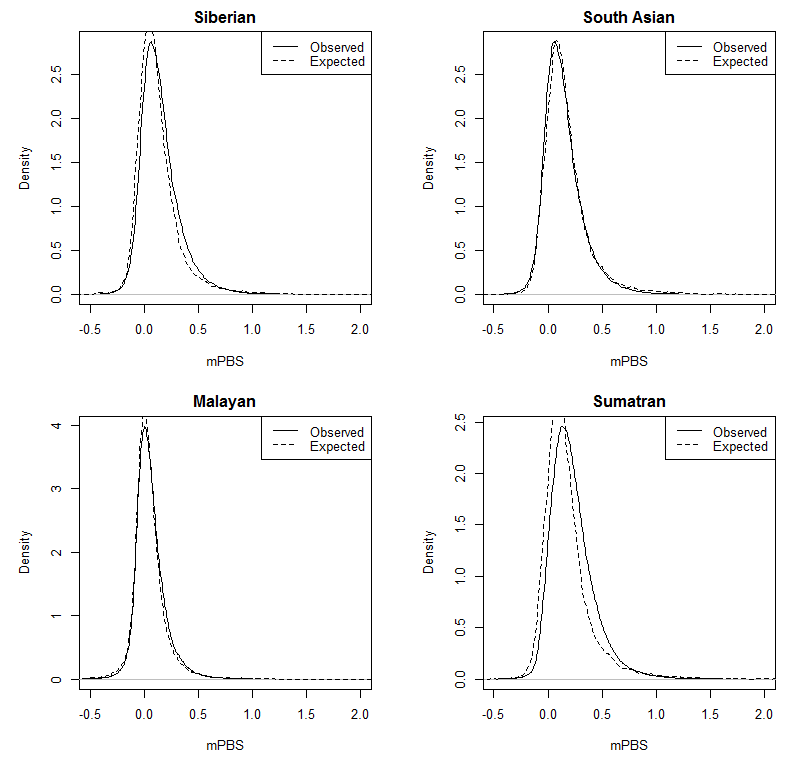


**Supplementary Figure 11**: Observed and expected mPBS distributions. For each population, we represented the observed against expected mPBS values obtained using simulations under a purely neutral model based on inferred demographic parameters. mPBS values have been computed using the same individuals as for demographic inference (Supplementary Table 7, Dataset 1).


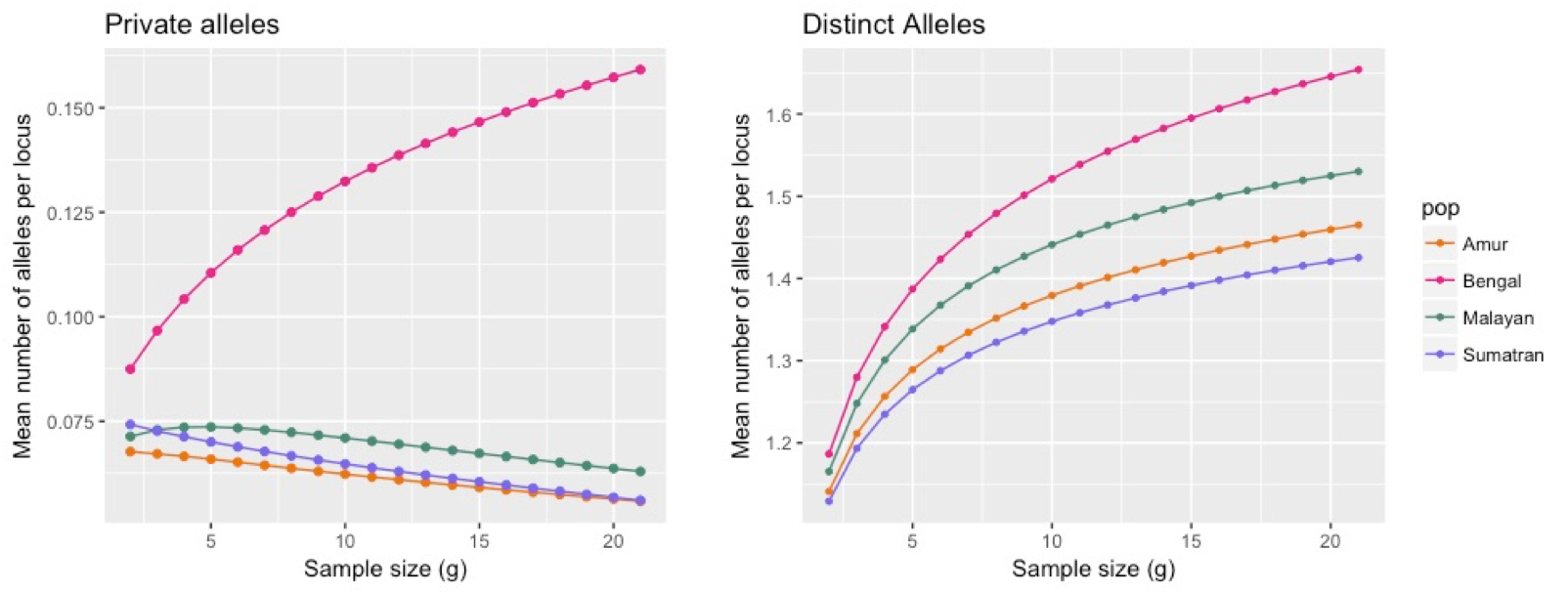


**Supplementary Figure 12**: ADZE plots of private alleles (alleles not found in other populations; left panel) and distinct (alleles unique to any given population, but allowing overlaps between populations).

**Supplementary Figure 13:** hPSMC plot of pairwise divergence among subspecies. Different pairs of population diverged between 9,000 and 20,000 years ago.**Supplementary tables**

**Supplementary Table 1**: List of tiger samples used within the coverage, in addition to sequencing information and associated AZA studbook numbers if associated. We used information from the tiger species survival plan and studbook to identify how the individuals we sampled were related to founders and specify in the table whether they were first (F1) or second generation (F2) descendants of founding/wild caught individuals.

| Map number | Sample ID | % bases covered | average depth | Source | Sample courtesy | SSB number | ancestry/location |
| --- | --- | --- | --- | --- | --- | --- | --- |
| 1 | AMU_ZOOK2 | 99 | 26.8784 | Captive | Published |  |  |
| 2 | AMU1 | 100 | 27.6484 | Captive | Omaha Zoological Society | 4203 | F1 |
| 3 | AMU2 | 100 | 26.6904 | Captive | San Diego Zoo | 260 | F1 |
| 4 | AMU3 | 91 | 4.43384 | Captive | Omaha Zoological Society | 3957 | F1 |
| 5 | AMU4 | 100 | 22.0467 | Wild | WCS Russia Program |  | Nezhinka river, Primorskii |
| 6 | AMU5 | 99 | 17.6047 | Wild | WCS Russia Program |  | Vyazemsky, Khabarovskii |
| 7 | AMU7 | 99 | 8.41758 | Wild | WCS Russia Program |  | Solontsovyi village, Khabarovskii |
| 8 | AMU8 | 99 | 14.387 | Wild | WCS Russia Program |  | Blagodatnoye, Primorskii |
| 9 | AMU9 | 100 | 19.2591 | Wild | WCS Russia Program |  | Blagodatnoye, Primorskii |
| 10 | AMU11 | 99 | 18.1544 | Wild | WCS Russia Program |  | Kunaleyka, Primorskii |
| 11 | AMU15 | 97 | 5.7804 | Captive | San Francisco Zoo | 4500 | F2 |
| 12 | AMU16 | 94 | 4.55855 | Captive | San Diego Zoo | 2686 | F2 |
| 13 | AMU17 | 96 | 5.39823 | Captive | San Diego Zoo | 5049 | F2 |
| 14 | AMU18 | 97 | 5.48226 | Captive | San Diego Zoo | 2879 | F2 |
| 15 | AMU20 | 100 | 27.6484 | Captive | Omaha Zoological Society | 2429 | F1 |
| 16 | AMU21 | 98 | 8.18089 | Captive | Omaha Zoological Society | 4013 | F2 |
| 17 | AMU22 | 96 | 5.92546 | captive | Omaha Zoological Society | 4187 | F2 |
| 18 | AMU23 | 94 | 4.8623 | captive | San Diego Zoo | 4638 | F2 |
| 19 | AMU24 | 100 | 17.1433 | Wild | WCS Russia Program |  | Nezhinka river, Primorskii |
| 20 | BEN_CI2 | 99 | 8.7746 | Wild | CCMB, Anuradha Reddy |  | Chandrapur |
| 21 | BEN_CI3 | 99 | 23.6797 | Wild | Wildlife Institute of India |  | Kanha Tiger Reserve |
| 22 | BEN_CI4 | 99 | 14.378 | Wild | Wildlife Institute of India |  | Kanha Tiger Reserve |
| 23 | BEN_CI5 | 99 | 14.6175 | Wild | Wildlife Institute of India |  | Kanha Tiger Reserve |
| 24 | BEN_CI6 | 99 | 18.9152 | Wild | Wildlife Institute of India |  | Kanha Tiger Reserve |
| 25 | BEN_CI7 | 99 | 9.33063 | Wild | Wildlife Institute of India |  | Kanha Tiger Reserve |
| 26 | BEN_CI8 | 90 | 5.23045 | Wild | CCMB, Anuradha Reddy |  | Pench Tiger Reserve |
| 27 | BEN_NE1 | 100 | 21.5036 | Wild | Aranyak |  | Assam |
| 28 | BEN_NE2 | 100 | 22.5455 | wild | Aranyak |  | Assam |
| 29 | BEN_NE3 | 99 | 14.8182 | wild | Aranyak |  | Assam |
| 30 | BEN_NOR1 | 99 | 22.7056 | wild | Wildlife Institute of India |  | Corbett Tiger Reserve |
| 31 | BEN_NOR2 | 100 | 23.6836 | wild | Wildlife Institute of India |  | Corbett Tiger Reserve |
| 32 | BEN_NW1 | 83 | 5.56259 | wild | Wildlife Institute of India |  | Ranthambore Tiger Reserve |
| 33 | BEN_NW3 | 100 | 21.6486 | wild | NCBS |  | Ranthambore Tiger Reserve |
| 34 | BEN_NW4 | 99 | 24.0216 | wild | NCBS |  | Ranthambore Tiger Reserve |
| 35 | BEN_SI1 | 100 | 20.5914 | wild | NCBS |  | Bandipur Tiger Reserve |
| 36 | BEN_SI2 | 99 | 9.14737 | wild | NCBS |  | Waynad Wildlife Sanctuary |
| 37 | BEN_SI3 | 100 | 21.6486 | wild | Kerala Forest Department |  | Waynad Wildlife Sanctuary |
| 38 | BEN_SI4 | 99 | 24.0216 | wild | Kerala Forest Department |  | Waynad Wildlife Sanctuary |
| 39 | BEN_SI5 | 99 | 13.0038 | wild | Kerala Forest Department |  | Periyar Tiger Reserve |
| 40 | BEN_SI6 | 100 | 18.3987 | wild | Kerala Forest Department |  | Waynad Wildlife Sanctuary |
| 41 | MAL1 | 100 | 32.8678 | wild caught | Omaha Zoological Society | 149 | Wild caught, Terenggan, Paka |
| 42 | MAL2 | 100 | 31.7846 | wild caught | El Paso zoo | 194 | Wild caught, Kelantan |
| 43 | MAL3 | 100 | 29.5029 | wild caught | San Diego Zoo | 196 | Wild caught, Kelantan |
| 44 | MAL4 | 100 | 21.9693 | wild caught | San Diego Zoo | 220 | Wild caught, Jeli |
| 45 | MAL5 | 93 | 4.19328 |  | San Diego Zoo | 37 | F1 |
| 46 | MAL6 | 96 | 4.74293 |  | San Diego Zoo | 184 | F2 |
| 47 | MAL7 | 95 | 4.99761 |  | WCS, Bronx Zoo | 190 | F2, sibling of 184 |
| 48 | MAL9 | 96 | 5.39176 |  | Omaha Zoological Society | 173 | F1, sibling of 66 |
| 49 | MAL10 | 97 | 6.18214 |  | San Diego Zoo | 168 | F2, sibling of 184 |
| 50 | MAL11 | 95 | 5.01651 |  | San Diego Zoo | 212 | F2 |
| 51 | MAL12 | 94 | 4.92575 |  | Omaha Zoological Society | 214 | F2, sibling of 212 |
| 52 | MAL13 | 96 | 5.91426 |  | San Diego Zoo | 66 | F1, sibling of 37 |
| 53 | MAL14 | 99 | 16.2318 |  | Omaha Zoological Society | 137 | Wild caught, Pehang |
| 54 | MAL15 | 97 | 6.14838 |  | San Diego Zoo | 67 | F2, sibling of 212 |
| 55 | MAL26 | 95 | 5.30186 |  | San Diego Zoo | 244 | F1 |
| 56 | SUM1 | 100 | 30.5978 |  | Omaha Zoological Society | 527 | F1 |
| 57 | SUM2 | 100 | 31.2564 |  | Omaha Zoological Society | 564 | F2 |
| 58 | SUM3 | 96 | 5.24071 |  | San Francisco Zoo | 722 | F2 |
| 59 | SUM4 | 91 | 4.45531 |  | San Diego Zoo | 311 | F2 |
| 60 | SUM5 | 95 | 4.74431 |  | San Diego Zoo | 381 | F2 |
| 61 | SUM6 | 100 | 21.6518 |  | San Diego Zoo | 1282 | F2 |
| 62 | SUM7 | 95 | 4.92926 |  | San Diego Zoo | 316 | F2, sibling of 311 |
| 63 | SUM8 | 97 | 6.3304 |  | San Diego Zoo | 1110 | F2 |
| 64 | SUM9 | 97 | 6.38643 |  | San Diego Zoo | 312 | F2, sibling of 311 |
| 65 | SUM13 | 99 | 14.8678 |  | San Diego Zoo | 768 | F2 |
| 66 | SUM14 | 96 | 5.75034 |  | San Diego Zoo | 1281 | F2, sibling of 1282 |

**Supplementary Table 2**: Assemblathon-2, repeat, annotation, and quality statistics of new genome compared with PanTig1.0 (Cho et al., 2013).

|  | Belahat 10x (Maltig1.0) | PanTig1.0 |
| --- | --- | --- |
| Contig N50 | 1.8 Mb | 39 kb |
| Contig L90 | 13,816 | 62,406 |
| Scaffold N50 | 21.3 Mb | 8.9 Mb |
| Scaffold L90 | 109 | 284 |
| % Scaffold Ns | 1.31% | 2.44% |
| Number of contigs | 36,444 | 126,370 |
| Number of scaffolds | 10,077 | 1,479 |
| % Repeats | 41.71% | 40.12% |
| Total genes annotated | 19,950 | 19,600 |
| % Complete BUSCOs | 92.9% | 84.7% |
| % Fragmented BUSCOs | 1.9% | 4.5% |
| % Duplicated BUSCOs | 0.4% | 0.1% |
| % Missing BUSCOs | 5.2% | 10.8% |

**Supplementary Table 3**: Details of analyses and samples used

| Analysis | SNP calling method | SNP Filters | Individuals used |
| --- | --- | --- | --- |
| **Population Clustering** |  |  |  |
| ADMIXTURE | freebayes | Q=30, GQ=30, DP=10, maf=0.025, max_miss=5% | ALL |
| PCA | freebayes | Q=30, GQ=30, DP=10, maf=0.025, max_miss=5% | ALL |
| NetStruct | freebayes | Q=30, GQ=30, DP=10, maf=0.025, max_miss=5% | ALL |
| FST | freebayes | Q=30, GQ=30, DP=10, maf=0.025, max_miss=5% | ALL |
| **Genetic Diversity** |  |  |  |
| SNV encounter rate | freebayes | Q=30, GQ=30, DP=10, maf=0.025, max_miss=5% | BEN_SI6, BEN_SI4, BEN_SI3, BEN_SI1, BEN_NW4, BEN_NW3, BEN_NOR1, BEN_NOR2, BEN_NE1, BEN_NE2, BEN_CI8, BEN_CI6, BEN_CI4, BEN_CI3, SUM13, SUM6, SUM2, SUM1, MAL14, MAL4, MAL3, MAL2, MAL1, AMU24, AMU11, AMU9, AMU5, AMU4, AMU2, AMU1 |
| ADZE | freebayes | Q=30, GQ=30, DP=10, maf=0.025, max_miss=5% | ALL |
| Nucleotide diversity | freebayes | Q=30, GQ=30, DP=10, maf=0.025, max_miss=5% | ALL |
| **Demographic history** |  |  |  |
| PSMC | bcftools | DP=10, maxDP=2*(average depth) | AMU1, MAL1, SUM2, BEN_SI3 |
| FastSimcoal (Global demographic history) | freebayes | Q=30, GQ=30, DP=10, regions within 50Kb of transcribing regions removed | SUM2, SUM1, SUM6, BEN_SI3, BEN_SI4, BEN_SI1, MAL1, MAL2, MAL3, MAL4, AMU2, AMU1, AMU4 |
| FastSimcoal (Bengal tiger demographic history) | freebayes | Q=30, GQ=30, DP=10, regions within 50Kb of transcribing regions removed | MAL1, MAL2, MAL3, BEN_CI3, BEN_CI6, BEN_NE2, BEN_NE1, BEN_NW4, BEN_NW3, BEN_SI3, BEN_SI4, BEN_SI1 |
| **Selection scans** |  |  |  |
| mPBS | freebayes | Q=30, GQ=30, DP=10 | BEN_SI6, BEN_SI5, BEN_SI4, BEN_SI3, BEN_SI1, BEN_NW4, BEN_NW3, BEN_NOR1, BEN_NOR2, BEN_NE1, BEN_NE2, BEN_NE3, BEN_CI6, BEN_CI5, BEN_CI4, BEN_CI3, SUM13, SUM6, SUM2, SUM1, MAL14, MAL4, MAL3, MAL2, MAL1, AMU24, AMU20,AMU11,AMU9, AMU8, AMU5, AMU4, AMU2, AMU1, AMU_ZOOK2 |
| **Inbreeding** |  |  |  |
| bcftools ROH | freebayes | Q=30, GQ=30, DP=10, maf=0.025, max_miss=5%, noSex | BEN_SI6, BEN_SI5, BEN_SI4, BEN_SI3, BEN_SI1, BEN_NW4, BEN_NW3, BEN_NOR1, BEN_NOR2, BEN_NE1, BEN_NE2, BEN_NE3, BEN_CI8, BEN_CI6, BEN_CI5, BEN_CI4, BEN_CI3, SUM13, SUM6, SUM2, SUM1, MAL14, MAL4, MAL3, MAL2, MAL1, AMU24, AMU20, AMU9, AMU5, AMU4, AMU2, AMU1, AMU_ZOOK2 |
| GARLIC | freebayes | Q=30, GQ=30, DP=10, maf=0.025, max_miss=5%, noSex | ALL |

**Supplementary Table 4**: Weighted F_ST_ between subspecies as computed by VCFtools.

| Population | Bengal | Malayan | Sumatran |
| --- | --- | --- | --- |
| Amur | 0.200 | 0.230 | 0.318 |
| Bengal |  | 0.164 | 0.242 |
| Malayan |  |  | 0.280 |

**Supplementary Table 5**: Weighted F_ST_ between putative Bengal sub-populations as computed by VCFtools. NOR=North India, SI=South India, NW=Northwest India, CI=Central India, NE=Northeast India.

|  | BEN_NOR | BEN_SI | BEN_NW | BEN_CI |
| --- | --- | --- | --- | --- |
| BEN_NE | 0.12752 | 0.18127 | 0.27993 | 0.11281 |
| BEN_NOR |  | 0.17404 | 0.29921 | 0.09356 |
| BEN_SI |  |  | 0.27524 | 0.12259 |
| BEN_NW |  |  |  | 0.20262 |

**Supplementary Table 6**: Selected samples and number of retained sites for the two datasets used in demographic inference.

|  | # SUM  inds | # BEN  inds | # MAL  inds | # AMU  inds | Samples | # polymorphic sites without filtering | # polymorphic sites after filtering |
| --- | --- | --- | --- | --- | --- | --- | --- |
| Dataset 1 | 3 | 3 | 4 | 3 | SUM2, SUM1, SUM6,  BEN_SI3, BEN_SI4, BEN_SI1, MAL1, MAL2, MAL3, MAL4, AMU2, AMU1, AMU4 | 2,892,663 | 265,560 |
| Dataset 2 | 0 | 9 | 3 | 0 | MAL1, MAL2, MAL3, BEN_CI3, BEN_CI6, BEN_NE2, BEN_NE1, BEN_SA4, BEN_SA3, BEN_SI3, BEN_SI4, BEN_SI1 | 2,935,205 | 267,933 |

**Supplementary Table 7**: Estimated demographic parameters and confidence intervals for the modeling of the four subspecies differentiation from an Asian metapopulation

| **Parameter acronyms** | **Parameter descriptions** | **Maximum likelihood estimate** | **lower  limit CI95%** | **upper limit CI95%** |
| --- | --- | --- | --- | --- |
| N_ANCEST | Ancestral Asian metapopulation size | 112,088 | 106,999 | 128,083 |
| N_ASIA | Asian metapopulation size | 8,281 | 6,443 | 15,953 |
| N_SUM | Sumatra population size | 5,380 | 3,765 | 11,892 |
| N_BEN | Bengal population size | 5,606 | 4,395 | 11,866 |
| N_MAL | Malayan population size | 12,801 | 7,338 | 13,392 |
| N_AMU | Amur population size | 9,735 | 3692 | 12,872 |
| TD_SUM | Sumatran divergence time | 9,147 | 6,378 | 9,650 |
| TD_BEN | Bengal divergence time | 11,842 | 9,900 | 13,795 |
| TD_MAL | Malayan divergence time | 7,775 | 2,820 | 8,490 |
| TD_AMU | Amur divergence time | 7,562 | 3,248 | 8,118 |
| IB_SUM | Sumatran initial bottleneck (founder effect) | 0.1241 | 0.0427 | 0.2329 |
| IB_BEN | Bengal initial bottleneck (founder effect) | 0.0827 | 0.0024 | 0.0987 |
| IB_MAL | Malayan initial bottleneck (founder effect) | 0.0032 | 0.0015 | 0.0179 |
| IB_AMU | Amur initial bottleneck (founder effect) | 0.0153 | 0.0023 | 0.0514 |
| RB_SUM | Sumatran recent bottleneck | 0.2035 | 0.1044 | 0.2959 |
| RB_BEN | Bengal recent bottleneck | 0.3106 | 0.3006 | 0.3777 |
| RB_MAL | Malayan recent bottleneck | 0.1204 | 0.0844 | 0.1457 |
| RB_AMU | Amur recent bottleneck | 0.2372 | 0.1768 | 0.2726 |
| Nm_SUM | SUM immigration rate | 0.1137 | 0.0004 | 0.2862 |
| Nm_BEN | BEN immigration rate | 3.1138 | 2.5340 | 4.7430 |
| Nm_MAL | MAL immigration rate | 0.0118 | 0.0008 | 1.0269 |
| Nm_AMU | AMU immigration rate | 0.2040 | 0.0008 | 1.1302 |
| IB_ANC | Ancestral Asian bottleneck | 1.0684 | 1.0156 | 1.2742 |
| T_BOT | Time of ancestral bottleneck | 234,238 | 196,525 | 245,795 |

Population sizes (N) are in number of diploid individuals assuming a mutation rate of 0.35e-8 (Liu et al. 2018). Times (T) are in years assuming a generation time of 5 years. Immigration rates (Nm) values are expressed as twice the number of diploids entering the population per generation, coming from the Asian metapopulation. Bottlenecks intensities (IB and RB) are indicated as bottleneck duration in units of 2N generations, but they were modeled as instantaneous bottlenecks (see fastsimcoal manual http://cmpg.unibe.ch/software/fastsimcoal2/man/fastsimcoal26.pdf, p. 27).

**Supplementary Table 8**: Estimated demographic parameters for the modeling of the four subspecies differentiation from an Asian metapopulation, without any divergence constraint

| **Parameter acronyms** | **Parameter descriptions** | **Maximum likelihood estimate** |
| --- | --- | --- |
| N_ANCEST | Ancestral Asian metapopulation size | 195,287 |
| N_ASIA | Asian metapopulation size | 43,326 |
| N_SUM | Sumatra population size | 11,216 |
| N_BEN | Bengal population size | 7,819 |
| N_MAL | Malayan population size | 10,629 |
| N_AMU | Amur population size | 8,201 |
| TD_SUM | Sumatran divergence time | 6,995 |
| TD_BEN | Bengal divergence time | 10,160 |
| TD_MAL | Malayan divergence time | 2,170 |
| TD_AMU | Amur divergence time | 3,850 |
| IB_SUM | Sumatran initial bottleneck (founder effect) | 0.003 |
| IB_BEN | Bengal initial bottleneck (founder effect) | 0.006 |
| IB_MAL | Malayan initial bottleneck (founder effect) | 0.008 |
| IB_AMU | Amur initial bottleneck (founder effect) | 0.010 |
| RB_SUM | Sumatran recent bottleneck | 0.36 |
| RB_BEN | Bengal recent bottleneck | 0.180 |
| RB_MAL | Malayan recent bottleneck | 0.130 |
| RB_AMU | Amur recent bottleneck | 0.230 |
| Nm_SUM | SUM immigration rate | 0.358 |
| Nm_BEN | BEN immigration rate | 0.180 |
| Nm_MAL | MAL immigration rate | 0.131 |
| Nm_AMU | AMU immigration rate | 0.226 |
| IB_ANC | Ancestral Asian bottleneck | 0.892 |
| T_BOT | Time of ancestral bottleneck | 225,815 |

Population sizes (N) are in number of diploid individuals assuming a mutation rate of 0.35e-8 (Liu et al. 2018). Times (T) are in years assuming a generation time of 5 years. Immigration rates (Nm) values are expressed as twice the number of diploids entering the population per generation, coming from the Asian metapopulation. Bottlenecks intensities (IB and RB) are indicated as bottleneck duration in units of 2N generations, but they were modeled as instantaneous bottlenecks (see fastsimcoal manual http://cmpg.unibe.ch/software/fastsimcoal2/man/fastsimcoal26.pdf, p. 27).

**Supplementary Table 9**: Estimated demographic parameters and confidence intervals for the modeling of four Bengal population differentiation from an Indian metapopulation

| **Parameter acronyms** | **Parameter descriptions** | **Maximum likelihood estimate** | **lower limit CI95%** | **upper limit CI95%** |
| --- | --- | --- | --- | --- |
| N_ANCEST | Ancestral Asian metapopulation size | 122,089 | 120,570 | 123,548 |
| N_META_IND | Indian metapopulation size | 7,987 | 7,459 | 11,208 |
| N_BEN_CI | CI population size | 4,481 | 3,099 | 11,440 |
| N_BEN_NE | NE population size | 11,525 | 5,208 | 13,160 |
| N_BEN_NW | NW population size | 4,742 | 2,026 | 11,575 |
| TD_BEN_CI | CI divergence time | 2,040 | 999 | 2,986 |
| TD_BEN_NE | NE divergence time | 6,784 | 5,924 | 8,109 |
| TD_BEN_NW | NW divergence time | 1,186 | 935 | 2,541 |
| TD_BEN_SI | SI divergence time | 1,548 | 971 | 2,429 |
| TD_BEN | Indian metapopulation divergence time | 8,462 | 8,056 | 9,110 |
| IB_BEN_CI | CI initial bottleneck (founder effect) | 0.0056 | 0.0025 | 0.0381 |
| IB_BEN_NE | NE initial bottleneck | 0.0166 | 0.0018 | 0.0644 |
| IB_BEN_SA | NW initial bottleneck | 0.3110 | 0.1137 | 0.4939 |
| IB_BEN_SI | SI initial bottleneck | 0.0078 | 0.0014 | 0.0119 |
| IB_META_IND | Indian metapopulation initial bottleneck | 0.0527 | 0.0364 | 0.0674 |
| RB_BEN_CI | CI recent bottleneck | 0.0042 | 0.0021 | 0.0375 |
| RB_BEN_NE | NE recent bottleneck | 0.0598 | 0.0028 | 0.0836 |
| RB_BEN_NW | NW recent bottleneck | 0.2076 | 0.0189 | 0.4141 |
| NM__Asia2IND | Indian immigration rate (from Asia) | 0.2031 | 0.0004 | 0.3015 |
| NM__BEN_CI | CI immigration rate | 0.0049 | 0.0010 | 1.1668 |
| NM__BEN_NE | NE immigration rate | 0.0104 | 0.0004 | 0.6793 |
| NM__BEN_NW | NW immigration rate | 0.2746 | 0.0004 | 0.7654 |
| NM__BEN_SI | SI immigration rate | 0.0103 | 0.0005 | 1.4335 |

Population sizes (N) are in number of diploid individuals, assuming a mutation rate of 0.35e-8 (Liu et al. 2018). Times (T) are in years, assuming a generation time of 5 years. Immigration rates (Nm) values are expressed as twice the number of diploids entering the population per generation, coming from the Asian metapopulation. Bottlenecks intensities (IB and RB) are indicated as bottleneck duration in units of 2N generations, but they were modeled as instantaneous bottlenecks (see fastsimcoal manual http://cmpg.unibe.ch/software/fastsimcoal2/man/fastsimcoal26.pdf, p. 27). The values of the following parameters BEN_SI were fixed to previously estimated values: N_BEN_SI=5606, RB_BEN_SI=0.3106.

**Supplementary Table 10**: Gene Ontology enrichment results. The 20 most significant GO terms are presented, as well as their total number of genes, the number of observed significant genes in a given term, the expected number of significant genes, the fold enrichment and the p-value of the Fisher’s exact test.


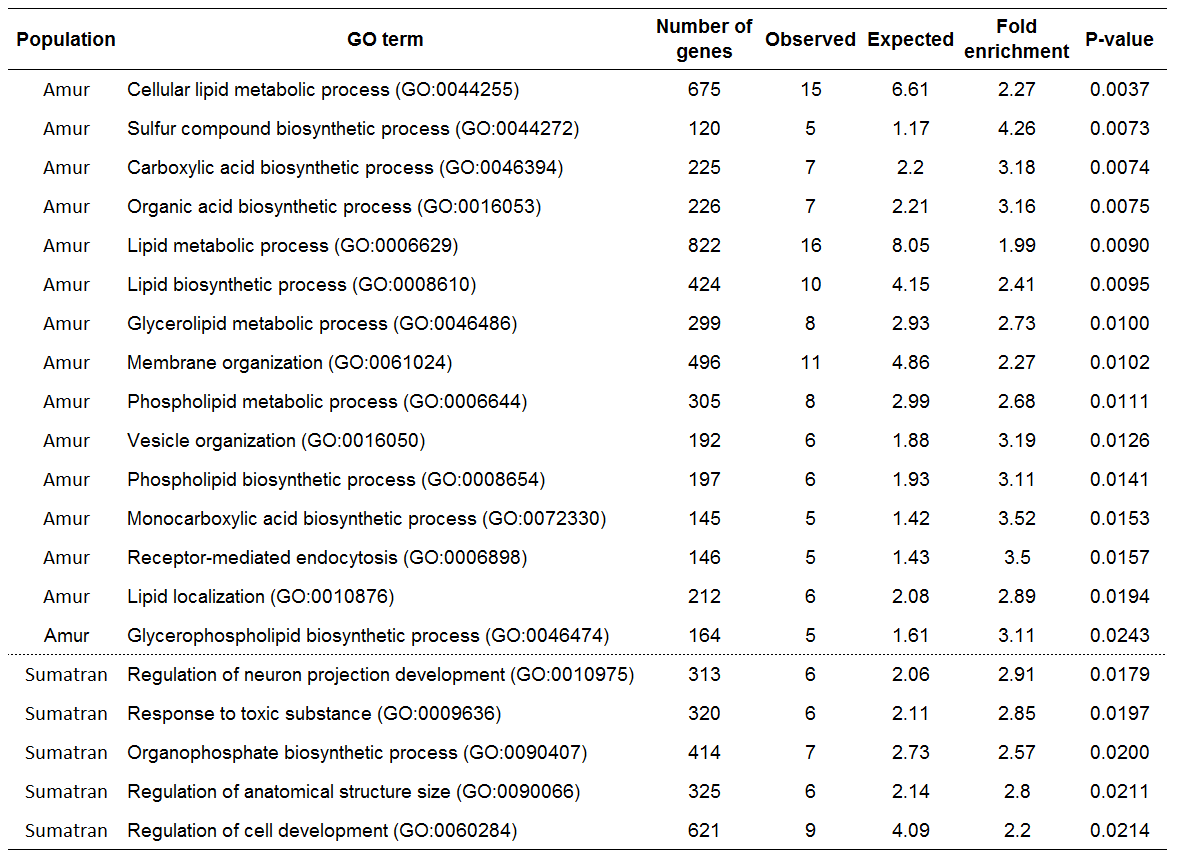


**Supplemental files**

Fastsimcoal input files used to estimate the Asian tiger population demography

| TIGERS8e.tpl |
| --- |
| //Simple model of tiger differentiation, no bottleneck  5 samples to simulate  //Population effective sizes  N_SUM  N_BEN  N_MAL  N_AMU  N_GHOST  //Samples sizes  6  6  8  6  0  //Growth rates : negative growth implies population expansion  0  0  0  0  0  //Number of migration matrices : 0 implies no migration between demes  2  //Migration matrix 0  0 0 0 0 0  0 0 0 0 0  0 0 0 0 0  0 0 0 0 0  0 0 0 0 0  //Migration matrix 1  0 0 0 0 MIG_SUM  0 0 0 0 MIG_BEN  0 0 0 0 MIG_MAL  0 0 0 0 MIG_AMU  0 0 0 0 0  //historical event: time, source, sink, migrants, new deme size, new growth rate, migration matrix index  16  0 0 0 0 1 0 0  50 0 0 0 RB_SUM 0 0 instbot  50 1 1 0 RB_BEN 0 0 instbot  50 2 2 0 RB_MAL 0 0 instbot  50 3 3 0 RB_AMU 0 0 instbot  50 0 0 0 1 0 1  TD_MAL 2 2 0 IB_MAL 0 1 instbot  TD_MAL 2 4 1 1 0 1  TD_AMU 3 3 0 IB_AMU 0 1 instbot  TD_AMU 3 4 1 1 0 1  TD_BEN 1 1 0 IB_BEN 0 1 instbot  TD_BEN 1 4 1 1 0 1  TD_SUM 0 0 0 IB_SUM 0 1 instbot  TD_SUM 0 4 1 1 0 1  TDMAX 4 4 0 R_ANC 0 0 //Ancestral population size change after last divergence  TANCBOT 4 4 0 IB_ANC 0 0 instbot //Ancestral inst bot possible speciation...  //Number of independent loci [chromosome]  1 0  //Per chromosome: Number of contiguous linkage Block: a block is a set of contiguous loci  1  //per Block:data type, number of loci, per generation recombination and mutation rates and optional parameters  FREQ 1 0 1e-8 OUTEXP |

| TIGERS9B3Mv1_r8e.est |
| --- |
| // Priors and rules file  // *********************  [PARAMETERS]  //#isInt? #name #dist.#min #max  //all Ns are in number of haploid individuals  //Population sizes -----------------------------------------  1 N_ANCEST unif 10000 100000 output REFERENCE //Ancestral size (meta pop size as well)  1 N_GHOST unif 1000 100000 output  1 N_SUM unif 1000 10000 output bounded  1 N_BEN unif 1000 10000 output bounded  1 N_MAL unif 1000 10000 output bounded  1 N_AMU unif 1000 10000 output bounded  //Divergence times -----------------------------------------  1 TD_SUM unif 400 1000 output bounded  1 TD_BEN unif 100 1000 output bounded  1 TD_MAL unif 100 1000 output bounded  1 TD_AMU unif 100 1000 output bounded  //Bottleneck intensities -----------------------------------  0 IB_SUM logunif 0.001 1 output  0 IB_BEN logunif 0.001 1 output  0 IB_MAL logunif 0.001 1 output  0 IB_AMU logunif 0.001 1 output  0 RB_SUM unif 0.01 1 output  0 RB_BEN unif 0.01 1 output  0 RB_MAL unif 0.01 1 output  0 RB_AMU unif 0.01 1 output  0 IB_ANC logunif 0.001 2 output  //Gene flow rates ------------------------------------------  0 Nm_SUM logunif 1e-4 5 output bounded  0 Nm_BEN logunif 1e-4 5 output bounded  0 Nm_MAL logunif 1e-4 5 output bounded  0 Nm_AMU logunif 1e-4 5 output bounded  //Ancestral population size change ------------------------  1 TPLUSANCBOT unif 1e3 1e5 hide  [RULES]  [COMPLEX PARAMETERS]  //Ancestral population size change ------------------------  1 MAX1 = TD_SUM%max%TD_BEN hide  1 MAX2 = MAX1%max%TD_MAL hide  1 TDMAX = MAX2%max%TD_AMU hide  1 TANCBOT = TDMAX+TPLUSANCBOT output  //Ancestral resize  0 R_ANC = N_ANCEST/N_GHOST hide  0 R_PREV = N_PREV/N_ANCEST hide  //Migration rates ------------------------------------------  0 MIG_SUM = Nm_SUM/N_SUM hide  0 MIG_BEN = Nm_BEN/N_BEN hide  0 MIG_MAL = Nm_MAL/N_MAL hide  0 MIG_AMU = Nm_AMU/N_AMU hide |

Note that we also performed a series of estimations by removing the upper limits on divergence times in the est file.

*Fastsimcoal input files used to estimate the Bengal tiger population demography conditional on estimated demography of Asian tigers*

Note that fixed parameter values taken from the previous estimation are rescaled parameters that take into account the mutation rate assumed in these estimations (1e-8 per site per generation, instead of 0.35 e-8 per site per generation used to compute estimated values reported in Supplementary Tables 7 and 8).

| TIGERS8e.tpl |
| --- |
| //Simple model of tiger differentiation, no bottleneck  9 samples to simulate  //Population effective sizes  3766  N_META_IND  8960  6814  N_GHOST_ASIA  N_BEN_CI  N_BEN_NE  N_BEN_SA  N_BEN_SI  //Samples sizes  0  0  6  0  0  4  4  4  6  //Growth rates : negative growth implies population expansion  0  0  0  0  0  0  0  0  0  //Number of migration matrices : 0 implies no migration between demes  2  //Migration matrix 0  0 0 0 0 0 0 0 0 0  0 0 0 0 0 0 0 0 0  0 0 0 0 0 0 0 0 0  0 0 0 0 0 0 0 0 0  0 0 0 0 0 0 0 0 0  0 0 0 0 0 0 0 0 0  0 0 0 0 0 0 0 0 0  0 0 0 0 0 0 0 0 0  0 0 0 0 0 0 0 0 0  //Migration matrix 1  0 0 0 0 3.018842e-05 0 0 0 0  0 0 0 0 m_Asia2IND 0 0 0 0  0 0 0 0 1.321606e-06 0 0 0 0  0 0 0 0 2.993084e-05 0 0 0 0  0 0 0 0 0 0 0 0 0  0 m_BEN_CI 0 0 0 0 0 0 0  0 m_BEN_NE 0 0 0 0 0 0 0  0 m_BEN_SA 0 0 0 0 0 0 0  0 m_BEN_SI 0 0 0 0 0 0 0  //historical event: time, source, sink, migrants, new deme size, new growth rate, migration matrix index  26  T_RB 0 0 0 0.2034946 0 0 instbot //Recent bottleneck in SUM  T_RB 2 2 0 0.1204263 0 0 instbot //Recent bottleneck in MAL  T_RB 3 3 0 0.237151 0 0 instbot //Recent bottleneck in AMU  T_RB 5 5 0 RB_BEN_CI 0 0 instbot //Recent bottlenecks in INDIA  T_RB 6 6 0 RB_BEN_NE 0 0 instbot  T_RB 7 7 0 RB_BEN_SA 0 0 instbot  T_RB 8 8 0 0.3105766 0 0 instbot  T_RB 0 0 0 1 0 1  TD_BEN_CI 5 5 0 IB_BEN_CI 0 1 instbot //Old bottlenecks at divergence time in INDIA  TD_BEN_CI 5 1 1 1 0 1  TD_BEN_NE 6 6 0 IB_BEN_NE 0 1 instbot  TD_BEN_NE 6 1 1 1 0 1  TD_BEN_SA 7 7 0 IB_BEN_SA 0 1 instbot  TD_BEN_SA 7 1 1 1 0 1  TD_BEN_SI 8 8 0 IB_BEN_SI 0 1 instbot  TD_BEN_SI 8 1 1 1 0 1  TD_META_IND 1 1 0 IB_META_IND 0 1 instbot  TD_META_IND 1 4 1 1 0 1  640 0 0 0 0.124119 0 1 instbot //Old bottleneck in SUM  640 0 4 1 1 0 1  544 2 2 0 0.003208539 0 1 instbot //Old bottlenecks in MAL  544 2 4 1 1 0 1  529 3 3 0 0.01528991 0 1 instbot //Old bottleneck in AMU  529 3 4 1 1 0 1  TDMAX 4 4 0 RES_ANC 0 0 //Ancestral population size change after last divergence  16397 4 4 0 1.068355 0 0 instbot //Instantaneous bottleneck in ancestral population  //Number of independent loci [chromosome]  1 0  //Per chromosome: Number of contiguous linkage Block: a block is a set of contiguous loci  1  //per Block:data type, number of loci, per generation recombination and mutation rates and optional parameters  FREQ 1 0 1.0e-8 OUTEXP |

| TIGERS9B3Mv1_r8e.est |
| --- |
| // Priors and rules file  // *********************  [PARAMETERS]  //#isInt? #name #dist.#min #max  //all Ns are in number of haploid individuals  //Population sizes -----------------------------------------  1 N_ANCEST unif 1000 50000 output REFERENCE //Ancestral size (meta pop size as well)  1 N_META_IND unif 5000 100000 output  1 N_BEN_CI unif 1000 10000 output bounded  1 N_BEN_NE unif 1000 10000 output bounded  1 N_BEN_SA unif 1000 10000 output bounded  1 N_BEN_SI unif 3924 3924 output bounded //Fixed to previously estimated value  1 N_GHOST_ASIA unif 5797 5797 hide //Fixed to previously estimated value  1 T_RB unif 57 57 hide //Fixed to rescaled time  //Divergence times -----------------------------------------  1 TD_META_IND unif 100 900 output bounded  0 TD_REL_BEN_CI unif 0 1 hide bounded  0 TD_REL_BEN_NE unif 0 1 hide bounded  0 TD_REL_BEN_SA unif 0 1 hide bounded  0 TD_REL_BEN_SI unif 0 1 hide bounded  //Bottleneck intensities -----------------------------------  0 RB_BEN_CI logunif 0.001 1 output  0 RB_BEN_NE logunif 0.001 1 output  0 RB_BEN_SA logunif 0.001 1 output  //0 RB_BEN_SI logunif 0.001 1 output  0 IB_BEN_CI logunif 0.001 1 output  0 IB_BEN_NE logunif 0.001 1 output  0 IB_BEN_SA logunif 0.001 1 output  0 IB_BEN_SI logunif 0.001 1 output  0 IB_META_IND logunif 0.001 1 output  //Gene flow rates ------------------------------------------  0 NM__Asia2IND logunif 1e-4 5 output  0 NM__BEN_CI logunif 1e-4 5 output  0 NM__BEN_NE logunif 1e-4 5 output  0 NM__BEN_SA logunif 1e-4 5 output  0 NM__BEN_SI logunif 1e-4 5 output  [RULES]  [COMPLEX PARAMETERS]  //Divergence times ----------------------------------------  1 TDS_META_IND = TD_META_IND-T_RB hide  1 TD_PLUS_BEN_CI = TD_REL_BEN_CI * TDS_META_IND hide  1 TD_PLUS_BEN_NE = TD_REL_BEN_NE * TDS_META_IND hide  1 TD_PLUS_BEN_SA = TD_REL_BEN_SA * TDS_META_IND hide  1 TD_PLUS_BEN_SI = TD_REL_BEN_SI * TDS_META_IND hide  1 TD_BEN_CI = TD_PLUS_BEN_CI + T_RB output  1 TD_BEN_NE = TD_PLUS_BEN_NE + T_RB output  1 TD_BEN_SA = TD_PLUS_BEN_SA + T_RB output  1 TD_BEN_SI = TD_PLUS_BEN_SI + T_RB output  1 TDMAX = TD_META_IND %max% 829 output  //Migration rates ------------------------------------------  0 m_Asia2IND = NM__Asia2IND/N_META_IND hide  0 m_BEN_CI = NM__BEN_CI/N_BEN_CI hide  0 m_BEN_NE = NM__BEN_NE/N_BEN_NE hide  0 m_BEN_SA = NM__BEN_SA/N_BEN_SA hide  0 m_BEN_SI = NM__BEN_SI/N_BEN_SI hide  //Ancestral resize  0 RES_ANC = N_ANCEST/N_GHOST_ASIA hide |
